# Effect of hyperthermic intraperitoneal chemotherapy on patients with advanced colorectal cancer: a systematic review and meta-analysis

**DOI:** 10.1186/s12957-025-04165-7

**Published:** 2026-01-29

**Authors:** Ziying Su, Yong Guo, Xiaosong Ru, Xiao Wang, Qiaoran Mao, Nuo Zhou, Zhili Xu, Luyi Huang, Chenyu Ge, Yaonan Hong, Fule He, Meilan Hu

**Affiliations:** 1 The First School of Clinical Medicine, Zhejiang Chinese Medical University, Hangzhou, China; 2 The First Affiliated Hospital, Zhejiang Chinese Medical University, Hangzhou, China; 3 Department of Traditional Chinese Medicine, Hebei General Hospital, Shijiazhuang, China; 4 Zhejiang Chinese Medicine Museum, Zhejiang Chinese Medical University, Hangzhou, China; 5 Department of Prevention and Health, Affiliated Hangzhou First People’s Hospital, School of Medicine, Westlake University, Hangzhou, China

**Keywords:** Colorectal cancer, Hyperthermic intraperitoneal chemotherapy, Survival, Recurrence, Complications

## Abstract

**Background:**

Advanced colorectal cancer (CRC) predisposes to peritoneal metastases (PM), leading to a decreased survival rate. Advanced CRC includes CRC with PM (CRC-PM) and locally advanced high-risk CRC without PM. The effectiveness of hyperthermic intraperitoneal chemotherapy (HIPEC) in prolonging survival and in treating or preventing PM after surgery for advanced CRC is still uncertain.

**Methods:**

A search of PubMed, Cochrane, Embase, and Web of science databases for relevant studies prior to April 2024 was performed. Data were analyzed using Stata/MP 17.0 software. The primary outcomes included overall survival (OS) and disease-free survival (DFS). Secondary outcomes were overall recurrence rate (ORR), PM rate, and complications. The quality of evidence was assessed using Grading of Recommendations, Assessment, Development, and Evaluations (GRADE).

**Results:**

A total of ten high-quality cohort studies and four randomized controlled trials (RCTs) were included, encompassing 2851 patients. HIPEC improved 1-year DFS (odds ratio (OR) = 1.64, 95%Cl: 1.09–2.46) and 5-year OS (OR = 1.49, 95%Cl: 1.10–2.03) in advanced CRC. HIPEC also reduced the overall PM rate (OR = 0.66, 95%Cl: 0.49–0.90). For advanced high-risk CRC without prior PM, HIPEC reduced the PM rate and had a preventive effect (OR = 0.71, 95%Cl: 0.52–0.97). In terms of complications, HIPEC increased the incidence of thrombopenia (OR = 5.77, 95%Cl: 1.65–20.09) and neutropenia (OR = 3.21, 95%Cl: 1.74–5.90). The quality of evidence ranged from high to very low.

**Conclusion:**

The use of HIPEC in treating advanced CRC may result in improved survival rates and a reduction in peritoneal recurrence or metastasis, although complications should be considered. Further investigation is required to clarify the role of HIPEC in more high-quality RCTs.

**Supplementary Information:**

The online version contains supplementary material available at 10.1186/s12957-025-04165-7.

## Introduction

The second highest percentage of cancer-related deaths worldwide is caused by colorectal cancer (CRC), which causes a serious burden on human society [1]. Between 2020 and 2024, the number of new CRC cases is projected to rise from 1.9 million to 3.2 million, with deaths increasing from 930,000 to 1.6 million [2]. The peritoneum is a common site of CRC metastasis [3]. The median survival of CRC-peritoneal metastases (CRC-PM) is 5.2 months. Even with treatment, the 5-year survival rate remains low [4, 5]. Therefore, CRC-PM has historically been regarded as a disease with limited treatment alternatives and a poor prognosis. Due to the limitations of routine imaging, the early detection of low-volume CRC-PM is challenging [6]. The patients are frequently diagnosed at a late stage, when symptoms have already manifested. Consequently, the difficulty of early diagnosis of PM makes it necessary to explore methods for preventing PM. Hyperthermic intraperitoneal chemotherapy (HIPEC) is employed to reduce microscopic residual lesions by delivering chemotherapeutic agents directly to the peritoneal cavity [7]. Advanced CRC includes CRC-PM and locally advanced high-risk CRC without PM. The role of HIPEC in advanced CRC is uncertain [8], which hampers the optimal management of CRC-PM.

The use of prophylactic HIPEC in locally advanced CRC is becoming increasingly prevalent. Its main goal is to prevent PM in patients at high risk [9]. Prophylactic HIPEC has been demonstrated to prolong survival and reduce the recurrence rate of gastric cancer (GC) in both the systemic and peritoneal regions after surgery [10]. However, the PROPHYLOCHIP-PRODIGE15 trial found that the secondary observation surgery combined with prophylactic HIPEC was unable to enhance disease-free survival (DFS) in PM high-risk CRCs [11]. Overall, the role of HIPEC as either a preventive or therapeutic approach in advanced CRC remains controversial, as different studies have produced inconsistent results [12, 13].

In a recent statement, the Peritoneal Surface Oncology Group International (PSOGI) emphasized the necessity for further investigation into the potential role of therapeutic and prophylactic HIPEC in CRC-PM [14]. To date, the relevant meta-analysis is underdeveloped. Outcome data from randomized controlled trials (RCTs) are limited and some are still ongoing. To explore the role of HIPEC in advanced CRC (CRC-PM and locally advanced high-risk CRC without previous PM), RCTs and high-quality cohort studies were included to comprehensively evaluate the effects of HIPEC in this meta-analysis.

## Methods

Pre-registration of the study protocol on the PROSPERO platform has been completed (registration number: CRD42024611405, https://www.crd.york.ac.uk/PROSPERO/view/CRD42024611405) [15]. The report follows the PRISMA declaration [16] and AMSTAR guidelines [17].

### Literature search strategy

PubMed, Cochrane, Web of Science, and Embase were the four databases we searched through in a systematic manner. The search strategy used medical subject headings (Appendix Table 1) and free text terms such as ‘HIPEC’ or ‘Hyperthermic Intraperitoneal Chemotherapy’ or “Hot Chemotherapy’ and ‘Rectal Neoplasms’ or ‘Colonic Neoplasms’ or ‘Colorectal Neoplasms’. The detailed search strategy is presented in Supplementary File 2. The study was conducted between April and May 2024 and the literature search was limited to the period before April 2024. We limited the language of the articles to English only. If two records belonged to the same clinical trial but reported separately on different outcomes, we also included the records to obtain additional data. For additional studies, the reference sections of pertinent review articles were also consulted.

### Criteria for inclusion and exclusion

Each of the included studies met the strict requirements of the PICOS (Population, Intervention, Comparison, Outcomes, and Study design) framework. Two authors (Z.S. and X.R.) independently screened the literature using EndNote software. Any disputes were resolved by third party discussion.

The specific criteria consisted of the following:

1. Population (P): Patients with advanced CRC undergoing potentially curative resection were included. In this study, the category of advanced CRC under investigation comprised CRC-PM and locally advanced CRC (stage T3-4) at high risk of PM. We excluded patients who had appendix cancer. In addition, patients who had undergone palliative surgery or non-peritoneal perfusion chemotherapy were excluded. The trial must clearly state whether or not the patient developed PM.
2. Intervention (I): Any kind of therapeutic or preventive HIPEC is eligible regardless of the agents, dosage and timing used. Both the intervention and control groups underwent potentially curative resection. However, only the intervention group received HIPEC.
3. Comparison (C): Curative operative strategies for managing advanced CRC.
4. Outcome (O): Overall survival (OS), DFS, the 5-year progression-free survival (PFS) were the primary endpoints. Secondary endpoints included the effect of HIPEC on overall and peritoneal recurrence, as well as complications. Complications included paralytic ileus, diarrhea, nausea or vomiting, anastomotic leakage, anastomotic hemorrhage, infection, and myelosuppressive conditions.
5. Study Design(S): RCTs and high-quality cohort studies were included. Conference abstracts, letters, conference papers, and other types of articles were not included, along with studies that were duplicates or lacked relevant outcome data.

### Evaluation of evidence quality

To evaluate RCTs’ potential risk of bias, the study employed the Cochrane Collaboration tool [18]. Risk levels were classified as either ‘low’, ‘high’ or ‘unknown’. The quality of the included RCTs was evaluated through an assessment of performance, selection, detection, reporting and attrition biases. A comprehensive assessment of the risk of bias in cohort studies was performed using the Newcastle-Ottawa Scale (NOS). The inclusion criteria stipulated that cohort studies must have an NOS score of at least 6 [19]. In the event of any disputes, third-party discussions were initiated to reach a resolution.

### Data extraction

Two researchers (Z.S. and X.W.) independently extracted information for each included study. The information was composed of the authors’ names, country, publication date, demographic characteristics and tumor staging of the target population, details of HIPEC implementation, and duration of follow-up. Furthermore, each study’s subgroups and primary outcomes were summarized. The sample size, number of events in the control and experimental groups for the relevant outcome were extracted for each study. In instances where the data was unavailable, we sought the assistance of the authors for obtaining unpublished data.

### Statistical analysis

We employed Stata/MP 17.0 software to statistically analyze the extracted data and chose odds ratio (OR) as the effect size for dichotomous variables. Prior to combining the effect sizes, the included studies were evaluated for heterogeneity to ascertain the extent of homogeneity among the studies. The Cochran’s Q test, the I^2^ statistic, and the degree of overlap between the confidence intervals (CI) were used to assess heterogeneity. A fixed effect model should be employed if the overlap of CI is large, *p* > 0.1, and I^2^ < 50%. Conversely, a small overlap of CI, *p* < 0.1 and I²> 50% indicate substantial heterogeneity between studies. A random effects model should be employed. Furthermore, we carried out subgroup analyses in order to exclude the interference of experimental design factors (RCT or NRCT) and explore the respective effects of therapeutic or preventive HIPEC on advanced CRC. In subgroup analyses, the included studies were categorized either by design (RCTs or NRCTs) or by HIPEC type—therapeutic HIPEC for CRC-PM (Treatment of peritoneal carcinomatosis) and prophylactic HIPEC for high-risk locally advanced CRC (Prophylaxis for peritoneal carcinomatosis). The detection of publication bias was carried out when the number of included papers for the primary and secondary outcomes reached 10.

### Quality of evidence

To assess the quality of the evidence, outcomes of overall effects were evaluated by Grading of Recommendations, Assessment, Development, and Evaluations (GRADE). The aggregation of these ratings results in an overall rating of certainty (very low, low, moderate, and high). For outcomes derived from both RCTs and NRCTs, the initial GRADE rating was determined according to the primary source of evidence. When RCTs predominated, the starting level of evidence was rated as high. When NRCTs predominated, it was rated as low. And the risk of bias was further adjusted based on the ROBINS-I assessment. If the proportions of RCTs and NRCTs were comparable, the quality of evidence from each type of study was summarized separately.

## Results

### Literature search and findings

**Fig. 1:**
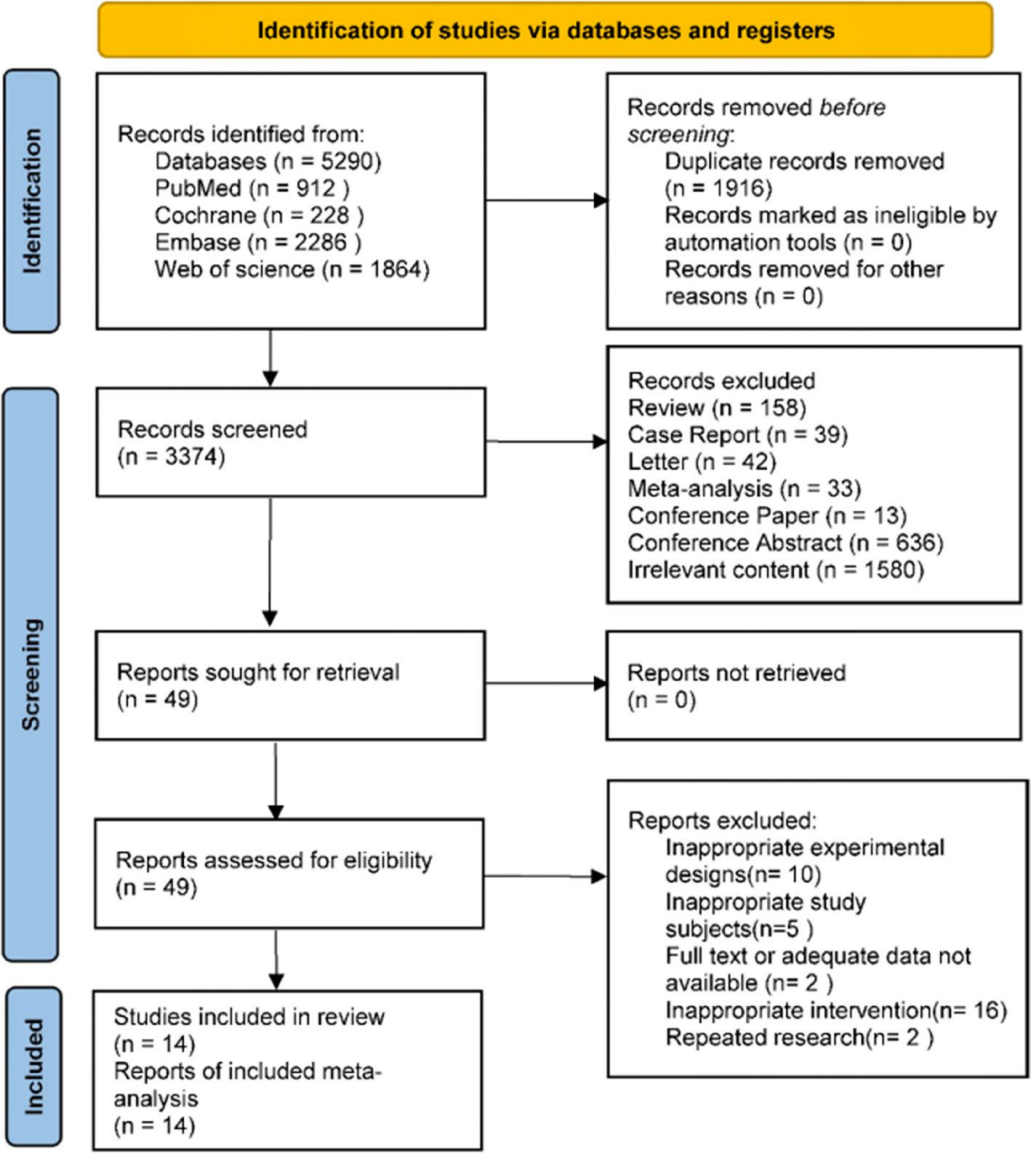
PRISMA flowchart

The two researchers (Z.S. and Q.M.) conducted independent literature searches. The preliminary search yielded 5,290 results. A total of 912, 228, 2286, and 1864 results were retrieved from the PubMed, Cochrane, Embase, and Web of Science databases, respectively. Duplicate studies were excluded, and irrelevant studies were removed through the reading of key content, including titles and abstracts. The aforementioned steps yielded 49 articles for comprehensive textual analysis. The remaining 35 articles were excluded because of deficiencies in their experimental design, the unsuitability of their study subjects, or the absence of pertinent data. Additionally, one study was a case-control study [20]. In order to reduce the impact of confounding factors introduced by differences in study design, these studies were excluded. Four RCTs and ten cohort studies were ultimately included. Dario Baratti et al. conducted various cohort studies in 2016 and 2019, and thus were not duplicates. Figure 1 illustrates the process of conducting a literature search.

### Risk of bias assessment

In RCTs and cohort studies, two researchers (Z.S. and N.Z.) independently assessed the risk of bias using the Cochrane Collaboration tool and the NOS tool, respectively. The RCTs clearly described the randomization methods. However, it remains unclear whether the allocation scheme was adequately concealed. Due to the unique characteristic of the research intervention, blinding investigators and subjects in the RCTs was unachievable. Consequently, the performance bias risk was classified as “high” while the detection bias risk remained “unclear” as none of the assessor’s blindness was described in the text. Appendix Fig. 1 clearly presents the outcomes of the bias risk evaluation conducted for RCTs. The NOS scores of all cohort studies exceeded 6, indicating a high quality. The results of the NOS scores are presented in Table 1.

**Table 1:** Summary of the Newcastle-Ottawa scale for assessing risk of bias in cohort studies

First Author (year)	Representativeness of the sample	Selection of non-exposed	Ascertainment of exposure	Outcome not present at start	Based on design and analysis	Assessment of outcome	Follow-up long enough for outcomes to occur	Adequacy of follow up of cohorts	Scores
Mo et al. (2024)[29]	*	*	*	*	**	*	*		8/9
Zheng et al. (2024)[34]	*	*	*	*	**	*	*		8/9
Guo et al. (2023)[28]	*	*	*	*	**	*	*		8/9
Li et al. (2021)[27]	*	*	*	*	**	*	*		8/9
Qin et al. (2022)[26]	*	*	*	*	**	*	*		8/9
Park et al. (2022)[30]	*	*	*	*	**	*	*	*	9/9
Sun et al. (2021)[27]	*	*	*	*	**	*	*		8/9
Baratti et al. (2019)[41]	*	*	*	*	**	*	*		8/9
Piso et al. (2018)[25]	*	*	*	*	*	*	*		7/9
Baratti et al. (2016)[31]	*	*	*	*	**	*	*		8/9

### Features of the study

Table 2 outlines the fourteen studies’ general characteristics. Table 3 shows the main results of each study. Of the included literature, four were RCTs involving a total of 651 subjects [21–24], while ten were cohort studies involving 2,200 subjects [25–34]. It should be noted that the studies conducted by Charlotte E. L. Klaver and Emma Sophia Zwanenburg were part of the same clinical trial. The former reported the primary endpoints and the latter reported the secondary endpoints. With regard to geographical location, seven studies were conducted in Asia and seven in Europe. China was the most frequently studied country, with six studies conducted there. For the presence or absence of PM, six clinical trials were conducted on subjects who did not have PM, and seven were conducted on subjects who had developed PM. In terms of HIPEC chemotherapy drugs, most studies used either mitomycin C or oxaliplatin, alone or in combination. Some also included additional agents such as cisplatin, lobaplatin, or raltitrexed. The HIPEC procedure was conducted within a temperature range of 42 to 43.5℃, with a duration that varied from 30 to 90 min. Additionally, CRS was conducted in seven studies.

**Table 2:** Characteristics of the studies included

Authors (Year)	Country	Tumor characteristics	Sample Size (I/C)	Gender (M/F)	Age	Surgery in both groups	Therapy regimen		follow-up (months)
							HIPEC	Control	
Zwanenburg et al. (2023)[24]	Netherlands	cT4N0–2M0/pT4N0–2M0 or perforated colon cancer	202 (102/100)	C: 52/50 I༚53/47	C:61(54–68) I:61(56–68)	resection of the primary tumor	Surgery + HIPEC (oxaliplatin (460 mg/m²), concurrent fluorouracil (400 mg/m²)/leucovorin (20 mg/m²) intravenously; temperature: 42–43℃; perfusion time: 30 min)	Surgery	59(54.5–64.5)
Klaver et al. (2019)[48]	Netherlands	cT4N0–2M0/pT4N0–2M0 or perforated colon cancer	202 (102/100)	C: 52/50 I༚53/47	C:61(54–68) I:61(56–68)	resection of the primary tumor	Surgery + HIPEC (oxaliplatin (460 mg/m²), concurrent fluorouracil (400 mg/m²)/leucovorin (20 mg/m²) intravenously; temperature: 42–43℃; perfusion time: 30 min)	Surgery	23(18–26)
Arjona-Sánchez et al. (2023)[9]	Spain	adenocarcinoma of the colon and rectum at stage cT4N0-2M0	184 (95/89)	C: 55/40 I༚56/33	C:62 ± 10.6 I:60 ± 8.7	extensive CRS and target surgery	Surgery + HIPEC (mitomycin C: 30 mg/m^2^; temperature: 42℃−43℃; perfusion time: over 60 min)	Surgery	36 (27–36)
Quénet et al. (2021)[21]	France	CRC-PM	265(132/133)	C: 67/65 I༚65/68	C:61(52–66) I:60(53–64)	CRS	Surgery + HIPEC (open: oxaliplatin (460 mg/m²); closed: oxaliplatin (360 mg/m²); temperature: 43℃; perfusion time: over 30 min)	Surgery	63.8(53.0–77.1)
Mo et al. (2024)[29]	China	pT3-4 locally advanced (pT3N + M0 and pT4NxM0) colon adenocarcinoma	927(788/139)	C: 473/315 I༚79/60	<60: C: 372; I: 75 ≥ 60: C: 416; I: 64	radical surgery	Surgery + HIPEC (1. lobaplatin: 50 mg/m^2^; flow rate: 1–2 L/min; temperature: 42–43℃; perfusion time: 45 min; 2. 5-FU: 600 mg/m^2^; flow rate: 400–600 mL/min; temperature: 42–43℃; perfusion time: 60–90 min, usually 60 min.)	Surgery	30 (22–41)
Zheng et al. (2024)[34]	China	cT4N0-2M0 CRC	180 (135/45)	C: 69/66 I༚23/22	≤ 40: C: 13; I: 8 ≥ 41: C: 122; I: 37	conventional laparotomy or laparoscopic radical surgery	Surgery + HIPEC (Mitomycin C (30–40 mg/m^2^) or Oxaliplatin (300 mg/m^2^); flow rate: 400 (± 100) ml/min; temperature: 43 (± 0.5) ℃; perfusion time: 60 min)	Surgery	52.0 (42.0–64.0)
Guo et al. (2023)[28]	China	colorectal adenocarcinoma (pT ≥ 4); no distant metastasis or PC	246 (123/123)	C: 77/46 I༚73/50	C:59.58 ± 12.910 I:58.49 ± 11.554	radical surgery	Surgery + HIPEC (drugs: mitomycin, lobaplatin, raltitrexed, or oxaliplatin; flow rate: 400 mL/min; temperature:43 ± 0.5℃; perfusion time: 60 min)	Surgery	C:20.28 ± 3.383 I:16.37 ± 3.431
Li et al. (2021)[27]	China	T4N0-2M0 colon adenocarcinoma	352(195/157)	C: 126/69 I༚90/67	C:58.5 ± 12.1 I:59.5 ± 9.9	radical resection	Surgery + HIPEC (closed; lobaplatin: 50mg/m^2^; flow rate:1–2 L/min; temperature:42–43℃; perfusion time: 45 min)	Surgery	C: 36 (26–45) I: 38 (29–47)
Qin et al. (2022)[26]	China	isolated synchronous colorectal peritoneal metastases	78 (35/43)	C: 19/16 I༚29/14	≤ 60: C: 21; I: 29 >60: C: 14; I: 14	CRS	Surgery + HIPEC (closed; 5-FU: 600mg/m^2^; flow rate: 400-600mL/min; temperature: 42℃; Perfusion time: 60 min)	Surgery	median = 46
Park et al. (2022)[30]	Republic of Korea	CRC-PM	100 (66/34)	C: 36/30 I༚19/15	C:63.6 ± 14.3 I:58.3 ± 11.7	CRS	Surgery + HIPEC (mitomycin C: 35 mg/m^2^; temperature: 42–43℃; Perfusion time: 90 min)	Surgery	C: median = 26.0 (range, 1–99) I: median = 25.5 (range, 5–98)
Sun et al. (2021)[27]	China	CRC-PM	82 (45/37)	C: 27/18 I༚21/16	C:61.6 ± 7.8 I:63.6 ± 7.0	palliative surgery or incomplete CRS	Surgery + HIPEC (oxaliplatin: 360 mg/m^2^; temperature: 42–43 °C; Perfusion time: 60 min)	Surgery	2 years
Baratti et al. (2019)[41]	Italy	CRC-PM	96 (48/48)	C: 21/27 I༚19/29	C:55.4(47.9–64.4) I:56.7(45.8–63.1)	CRS	Surgery + HIPEC (closed; mitomycin C (3.3 mg/m^2^/L) plus cisplatin (25 mL/m^2^/L), or mitomycin C alone (35 mg/m^2^); temperature: 42.5℃; Perfusion time:60 min)	Surgery	C: median = 31.6 I: median = 39.9
Piso et al. (2018)[25]	Germany	CRC-PM	73 (45/28)	C: 19/26 I༚14/14	<60: C: 11; I: 17 ≥ 60: C: 34; I:11	CRS	Surgery + HIPEC(mitomycin C: 60 min; oxaliplatin: 30 min)	Surgery	median = 53.8 mean = 56.9
Baratti et al. (2016)[31]	Italy	CRC at high risk for metachronous PM (with minimal synchronous PM)	66 (44/22)	C: 19/25 I༚8/14	C:57.5 ± 13.1 I:57.1 ± 10.7	Primary curative surgery	Surgery + HIPEC (closed; cisplatin (25 mg/m^2^/L) and mitomycin-C (3.3 mg/m^2^/L); temperature: 42.5 °C; Perfusion time: 60 min)	Surgery	C: median = 34.5 (95% CI: 21.1–47.9) I: median = 65.2 (95% CI: 50.9–79.5)

Data are expressed as mean ± SD, or median (interquartile range)

*C* control, *CI* confidence interval, *CRC* colorectal cancer, *CRS* cytoreductive surgery, *DFS* disease-free survival, *HIPEC* hyperthermic intraperitoneal chemotherapy, *I* intervention, *OS* overall survival, *PC* peritoneal carcinomatosis, *PFS* progression-free survival, *PM* peritoneal metastases

**Table 3:** Summary of main findings

Authors	RCT/ NRCT	Group	Number of participants	OS/DFS (months)	DFS (%)	OS (%)				Recurrence (%)	
						1-year	2-year	3-year	5-year	Overall	Peritoneal
Mo et al.	NRCT	Surgery + HIPEC	139							20.1	12.9
		Surgery	788							20.9	8.8
Qin et al.	NRCT	Surgery + HIPEC	43						66.8		
		Surgery	35						31.2		
Sun et al.	NRCT	Surgery + HIPEC	37	Mean OS: 11.4 ± 3.2, 95% CI: 7.7–9.9							
		Surgery	45	Mean OS: 9.5 ± 3.8, 95% CI: 8.3–10.6							
Li et al.	NRCT	Surgery + HIPEC	157	Median DFS: 44.5, 95% CI: 40.9–48.2.9.2 Median OS: 52.0, 95% CI: 48.9–55.1	The 1-year DFS: 93.3 The 3-year DFS: 61.1	100		82.7		32.5	26.1
		Surgery	195	Median DFS: 40.0, 95% CI: 32.4–47.6.4.6 Median OS: 63.0, 95% CI: 54.4–71.6	The 1-year DFS: 89.3 The 3-year DFS: 51.7	100		76.9		42.6	37.4
Piso et al.	NRCT	Surgery + HIPEC	28	Median OS: 41.6			79.2		23.5		
		Surgery	45	Median OS: 11.4			33.6		12.6		
Zheng et al.	NRCT	Surgery + HIPEC	45	Mean DFS: 74.13, 95% CI: 67.35–80.92	The 3-year DFS: 83.90						2.2
		Surgery	135	Mean DFS: 61.27, 95% CI: 55.87–66.66	The 3-year DFS: 70.10						11.1
Park et al.	NRCT	Surgery + HIPEC	34								
		Surgery	66								
Guo et al.	NRCT	Surgery + HIPEC	123				82.0				
		Surgery	123				88.5				
Baratti et al.(2016)[31]	NRCT	Surgery + HIPEC	22						81.3		9.3
		Surgery	44						70.0		42.5
Baratti et al.(2019)[32]	NRCT	Surgery + HIPEC	48	Median OS: 34.8					33.9		
		Surgery	48	Median OS: 39.3					21.6		
Zwanenburg et al.	RCT	Surgery + HIPEC	100		The 5-year DFS: 55.7				69.6		
		Surgery	102		The 5-year DFS: 52.3				70.9		
Klaver et al.	RCT	Surgery + HIPEC	100		The 18-month DFS: 69.0						10.3
		Surgery	102		The 18-month DFS: 69.3						22.5
Quénet et al.	RCT	Surgery + HIPEC	133	Median OS: 41.7, 95% CI: 36.2–53.8		86.9			39.4		
		Surgery	132	Median OS: 41.2, 95% CI: 35.1–49.7		88.3			36.7		
Arjona-Sánchez et al.	RCT	Surgery + HIPEC	89		The 3-year DFS: 81.2			91.7			2.2
		Surgery	95		The 3-year DFS: 78.0			92.9			10.5

Data are presented as mean ± SD. *RCT* randomized controlled trials, *NRCT* nonrandomized studies

### Primary outcome

#### Overall survival (Fig. 2)

##### Overall 1-year survival

François Quénet and T. Li reported 1-year survival [21, 33]. T. Li observed a 100% 1-year survival rate across both the experimental and comparison groups. Therefore, ORs and forest plots could not be generated. Differently, François Quénet’s observations in the PRODIGE7 trial revealed that the surgery group achieved an 88.3%, while the HIPEC + surgery group attained an 86.9%.

**Fig. 2:**
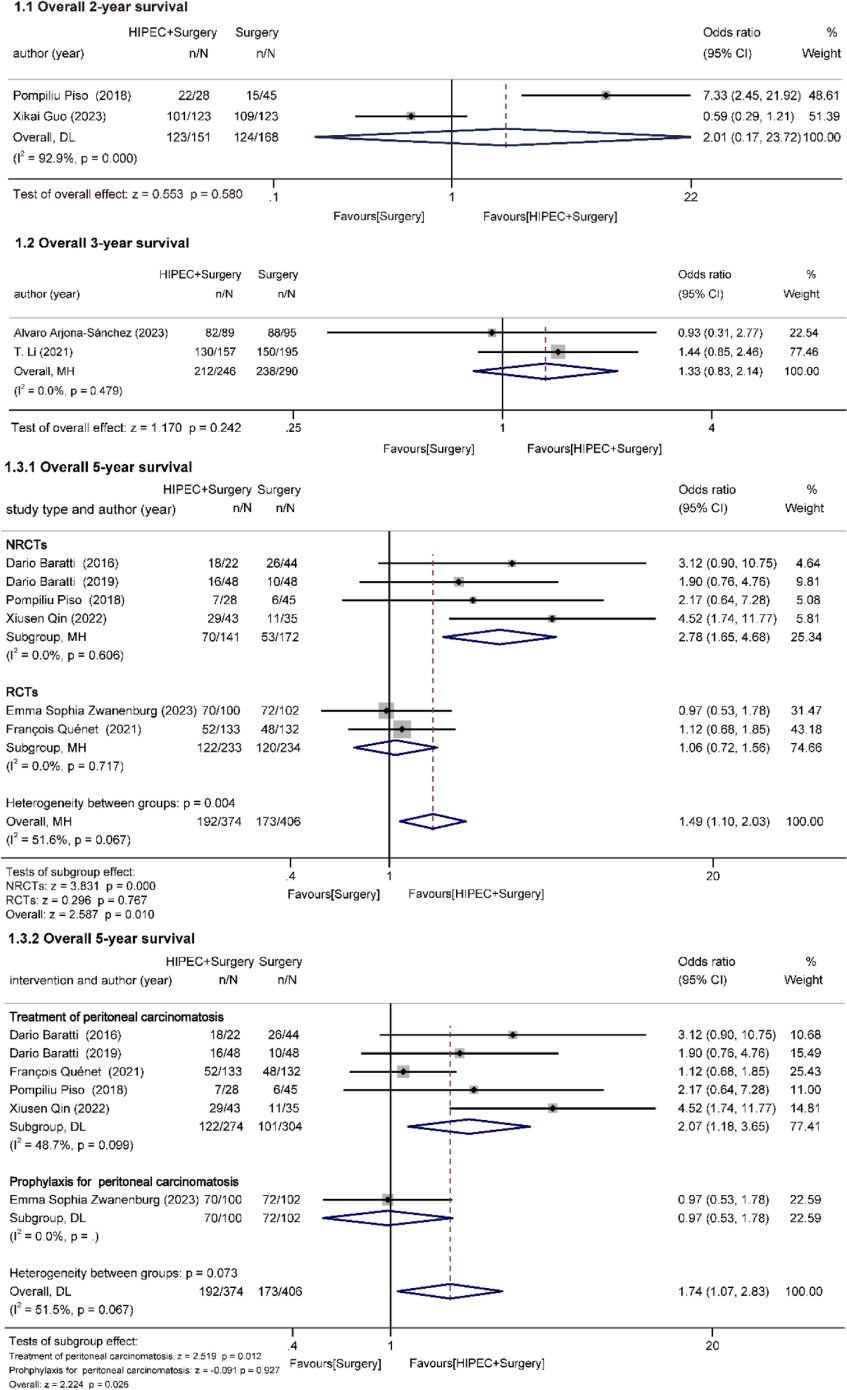
Effect of HIPEC on overall survival

##### Overall 2-year survival

Pompiliu Piso and Xikai Guo (two cohort studies) reported 2-year survival [25, 28]. 151 patients underwent HIPEC + surgery and 168 patients underwent surgery. Statistical analyses showed large inter-study heterogeneity (I^2^ = 92.9%, *P* = 0.000), which may be related to the different types and doses of drugs employed for HIPEC. Statistical analysis showed that HIPEC had no impact on 2-year survival in advanced CRC (OR = 2.01, 95%CI: 0.17–23.72, z = 0.553, *p* = 0.580 > 0.05).

##### Overall 3-year survival

Alvaro Arjona-Sánchez and T. Li (one RCT, one cohort study) reported 3-year survival [22, 33]. 246 patients underwent HIPEC + surgery and 290 patients underwent surgery. No heterogeneity was identified among the studies (I^2^ = 0.0%, *P* = 0.479). The results suggested that HIPEC did not have an effect on 3-year survival in advanced CRC (OR = 1.33, 95%Cl: 0.83–2.14, z = 1.170, *p* = 0.242 > 0.05).

##### Overall 5-year survival

A total of six studies (two RCTs, four cohort studies) reported the effect of HIPEC on 5-year survival [21, 24–26, 31, 32]. 374 patients underwent HIPEC + surgery and 406 patients underwent surgery. Statistical analyses showed large inter-study heterogeneity (I^2^ = 51.6%, *p* = 0.067). The overall results suggested that HIPEC significantly improved 5-year survival in advanced CRC (OR = 1.49, 95%Cl: 1.10–2.03, z = 2.587, *p* = 0.010 < 0.05). To look for sources of heterogeneity, further subgroup analyses were conducted. Heterogeneity was within acceptable limits after grouping according to the presence or absence of PM (I^2^ = 48.7%, *P* = 0.099). The results revealed that the utilization of HIPEC for CRC-PM was associated with an improved 5-year survival rate compared with the control group (OR = 2.07, 95%Cl: 1.18–3.65, z = 2.519, *p* = 0.012 < 0.05). In addition, grouped by study category, there was no heterogeneity between the NRCT and RCT groups. The RCT group showed no significant effect of HIPEC (OR = 1.06, 95%Cl: 0.72–1.56, z = 0.296, *p* = 0.767 > 0.05). Whereas, a statistically significant 5-year survival benefit in favor of the HIPEC group was seen in the NRCT group (OR = 2.78, 95%Cl: 1.65–4.68, z = 3.381, *p* = 0.000 < 0.05).

#### Disease-free survival (Fig. 3)

##### The 1-year disease-free survival

François Quénet and T. Li (one RCT, one cohort study) reported 1-year DFS (RFS combined into DFS) [21, 33]. 290 patients underwent HIPEC + surgery and 327 patients underwent surgery. No heterogeneity was identified among the studies (I^2^ = 0.0, *P* = 0.948). The analysis indicated that HIPEC improved 1-year DFS in advanced CRC (OR = 1.64, 95%Cl: 1.09–2.46, z = 2.354, *p* = 0.019 < 0.05).

**Fig. 3:**
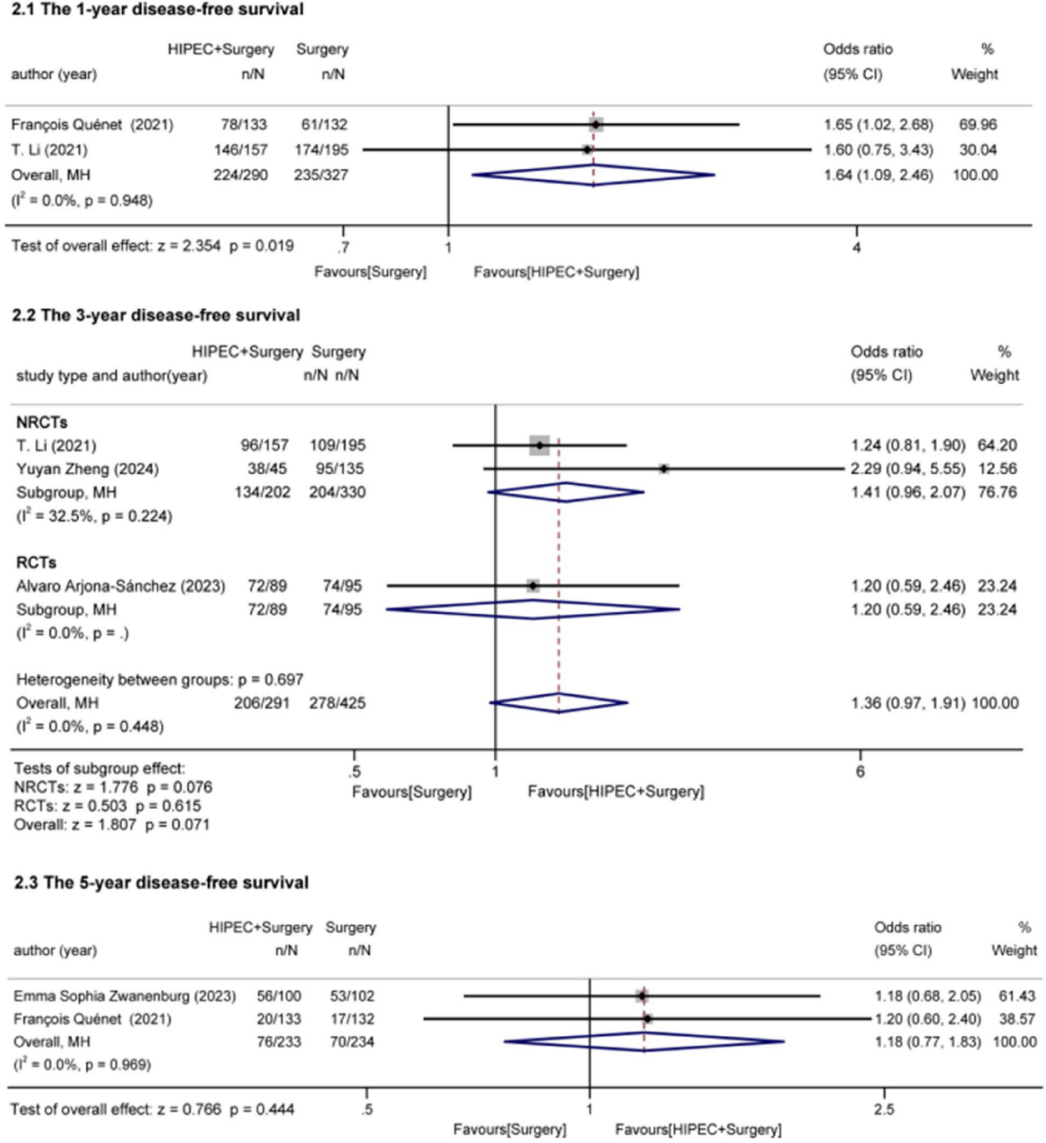
Effect of HIPEC on disease-free survival

##### The 3-year disease-free survival

Three studies (one RCT, two cohort studies) reported 3-year DFS regarding prophylactic HIPEC in advanced CRC without PM[22, 33, 34]. 291 patients received HIPEC + surgery, and 425 patients received surgery. No heterogeneity was identified among the studies (I²=0.0, *P* = 0.448). Neither the NRCT group (OR = 1.41, 95%Cl: 0.96–2.07, z = 1.776, *p* = 0.076 > 0.05) nor the RCT group (OR = 1.20, 95%Cl: 0.59–2.46, z = 0.503, *p* = 0.615 > 0.05) showed a significant effect of HIPEC.

##### The 5-year disease-free survival

Two RCT studies reported 5-year DFS [21, 24]. 233 patients received HIPEC + surgery, and 234 patients received surgery. No heterogeneity was identified among the studies (I^2^ = 0.0, *p* = 0.969). The analysis indicated that HIPEC did not significantly affect 5-year DFS (OR = 1.18, 95%Cl = 0.77–1.83, z = 0.766, *p* = 0.444 > 0.05).

#### The 5-year progression-free survival (Fig. 4)

Dario Baratti and François Quénet (one RCT, one cohort study) reported 5-year PFS of HIPEC for advanced CRC with PM[21, 31]. 155 patients received HIPEC + surgery, and 176 patients received surgery. Statistical analyses showed large inter-study heterogeneity (I^2^ = 91.6%, *p* = 0.001), possibly due to differences in HIPEC drug, dose, or type of study. The results showed no significant effect of HIPEC on 5-year PFS (OR = 2.80, 95%Cl: 0.28–28.39, z = 0.871, *p* = 0.384 > 0.05).

**Fig. 4:**
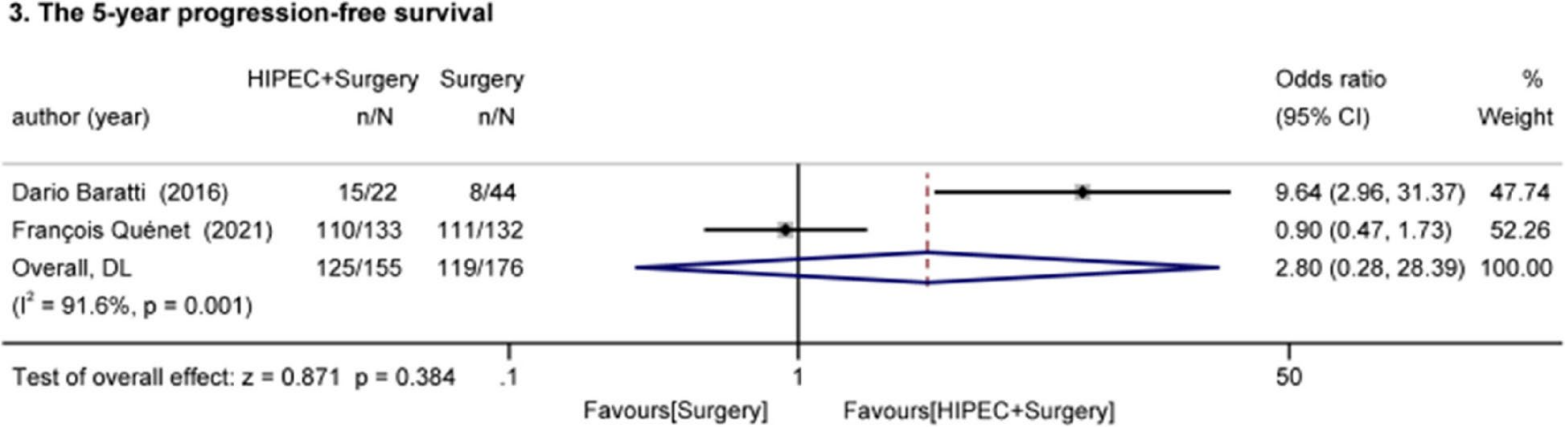
Effect of HIPEC on the 5-year progression-free survival

### Secondary outcomes

#### Recurrence rate

##### Overall recurrence rate (Fig. 5)

Overall recurrence rate (ORR) was assessed in five studies (two RCTs, three cohort studies) [21, 22, 26, 29, 33]. 561 patients underwent HIPEC + surgery and 1,245 patients underwent surgery. No heterogeneity was identified among the studies (I^2^ = 0.0%, *p* = 0.464). Overall results showed no significant effect of HIPEC on ORR (OR = 0.82, 95%Cl: 0.64–1.05, z=−1.570, *p* = 0.116 > 0.05). Neither the NRCT group (OR = 0.82, 95% CI: 0.61–1.11, z=−1.256, *p* = 0.209 > 0.05) nor the RCT group (OR = 0.81, 95% CI: 0.52–1.26, z=−0.947, *p* = 0.343 > 0.05) showed a significant effect of HIPEC. Similarly, the statistical results indicated that both prophylactic HIPEC (OR = 0.75, 95% Cl: 0.56–1.01, z=−1.865, *p* = 0.062 > 0.05) and therapeutic HIPEC (OR = 1.01, 95% Cl: 0.63–1.62, z = 0.053, *p* = 0.958 > 0.05) had no notable impact on the ORR.

**Fig. 5:**
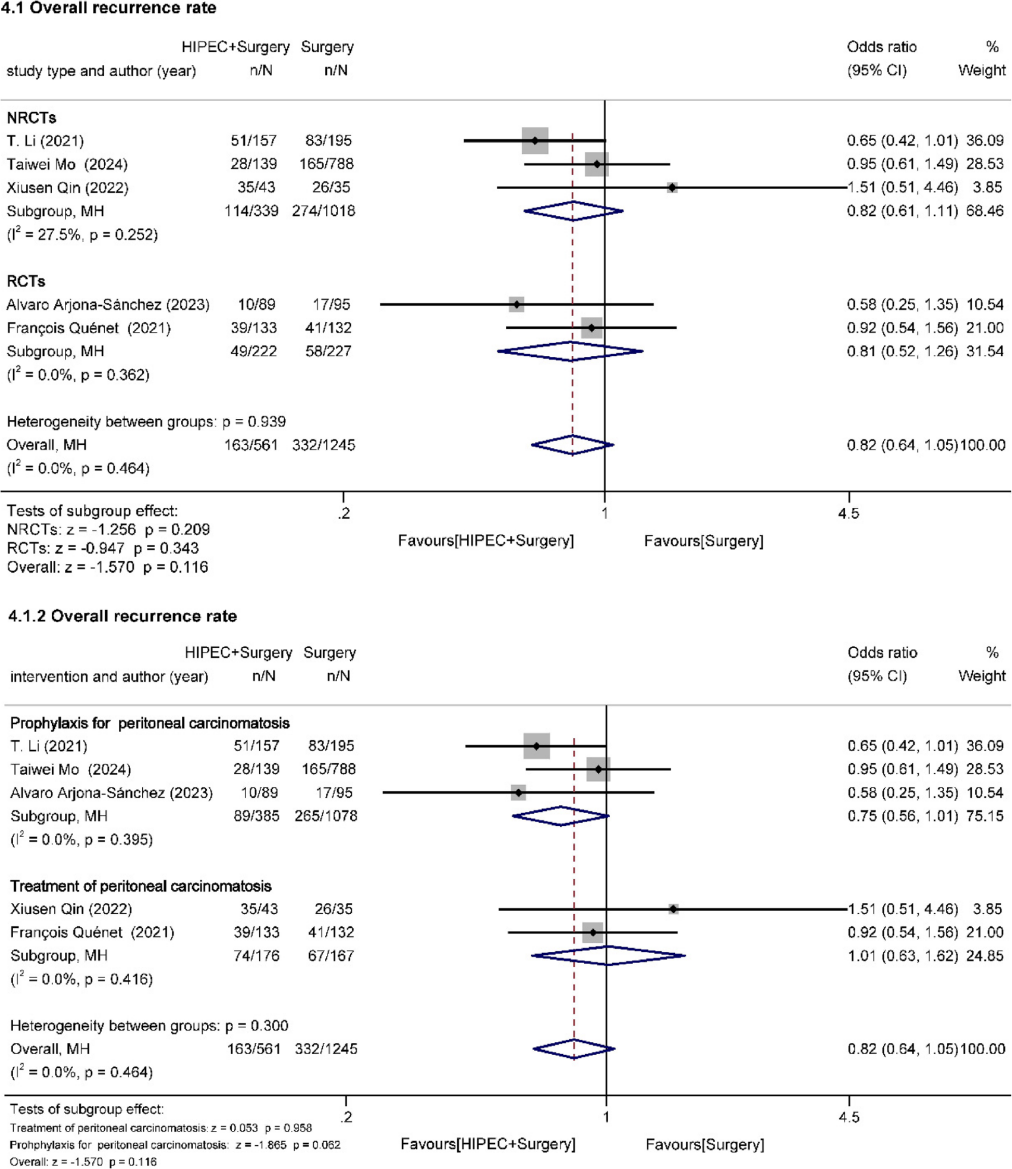
Effect of HIPEC on overall recurrence rate

##### Peritoneal metastasis rate (Fig. 6)

PM rate was evaluated in six studies (two RCTs, four cohort studies) [22, 23, 29, 31, 33, 34]. 539 patients underwent HIPEC + surgery and 1359 patients underwent surgery. There was a high degree of inter-study heterogeneity (I^2^ = 73.3%, *p* = 0.002). The analysis indicated that HIPEC significantly reduced the overall PM rate (OR = 0.66, 95%Cl: 0.49–0.90, z=−2.605, *p* = 0.009 < 0.05). The results of the NRCT group (OR = 0.77, 95%Cl: 0.55–1.08, z=−1.526, *p* = 0.127 > 0.05) suggested no statistical difference, whereas the RCT group showed that HIPEC significantly reduced PM rate in advanced CRC (OR = 0.34, 95%Cl: 0.16–0.70, z=−2.900, *p* = 0.004 < 0.05). In addition, there was some heterogeneity in the prophylactic HIPEC subgroup (I^2^ = 72.4%, *p* = 0.006). However, the statistical results showed that in advanced CRC without PM, HIPEC could significantly reduce the rate of PM and play a role in preventing PM (OR = 0.71, 95%Cl: 0.52–0.97, z=−2.151, *p* = 0.032 < 0.05).

**Fig. 6:**
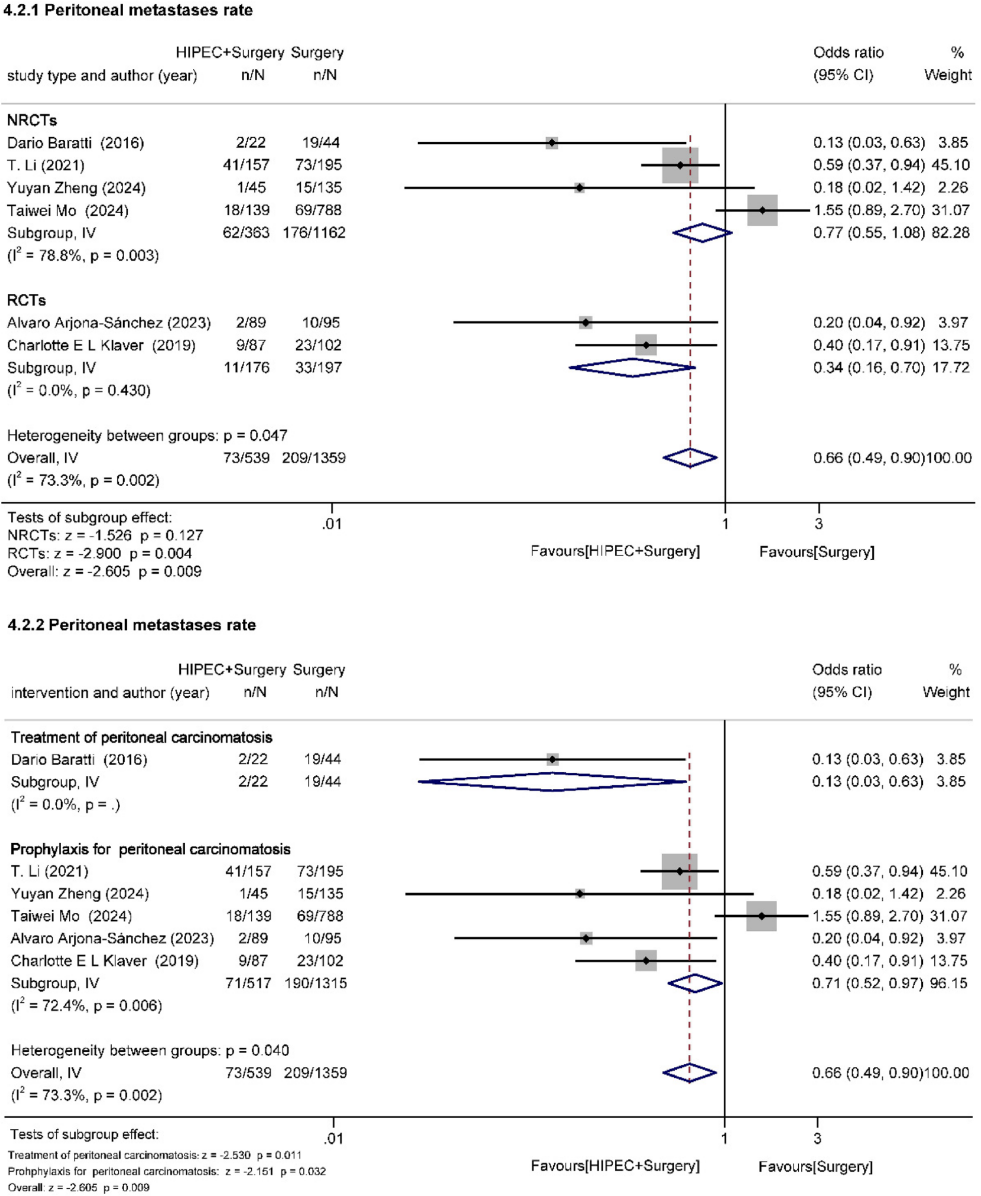
Effect of HIPEC on peritoneal metastasis rate

##### Complications

Major complications of HIPEC, including bleeding, intestinal perforation, bowel obstruction, and death, are uncommon [35]. We analyzed HIPEC-related complications that were reported at least twice in the included studies. Therefore, some of the complications listed below may not represent major HIPEC complications. All available data on relevant complications were included in the pooled analysis.

The results showed that complications regarding paralytic ileus (Appendix Fig. 2), diarrhea (Appendix Fig. 3), nausea or vomiting (Appendix Fig. 3), anastomotic leakage or hemorrhage (Appendix Fig. 4), wound infection (Appendix Fig. 5), abdominal infection (Appendix Fig. 5), lung infection (Appendix Fig. 5), anemia (Appendix Fig. 6) were not significantly different before and after HIPEC. However, we found that HIPEC significantly increased the incidence of thrombopenia and neutropenia.

Thrombopenia (Appendix Fig. 6) was evaluated in two RCTs [21, 22]. 222 patients underwent HIPEC + surgery and 227 patients underwent surgery. No heterogeneity was identified among the studies (I^2^ = 0.0%, *p* = 0.783). Overall results showed that thrombopenia was significantly favorable to surgery (OR = 5.77, 95%Cl: 1.65–20.09, z = 2.751, *p* = 0.006 < 0.05).

Neutropenia (Appendix Fig. 7) was evaluated in three studies (two RCTs, one cohort study) [21, 22, 27]. A total of 259 patients underwent HIPEC + surgery, and 272 operated without HIPEC. No heterogeneity was identified among the studies (I^2^ = 0.0%, *p* = 0.563). Overall, HIPEC significantly increased the occurrence of neutropenia (OR = 3.21, 95%Cl: 1.74–5.90, z = 3.749, *p* = 0.000 < 0.05). The RCT group’s results also yielded a notable impact of HIPEC on the incidence of neutropenia (OR = 2.80, 95%Cl: 1.39–5.64, z = 2.871, *p* = 0.004 < 0.05). As for patients with PM, the statistical difference also existed (OR = 2.98, 95%Cl: 1.54–5.79, z = 3.231, *p* = 0.001 < 0.05).

#### Effect of intraoperative or postoperative HIPEC on peritoneal metastasis rate

**Fig. 7:**
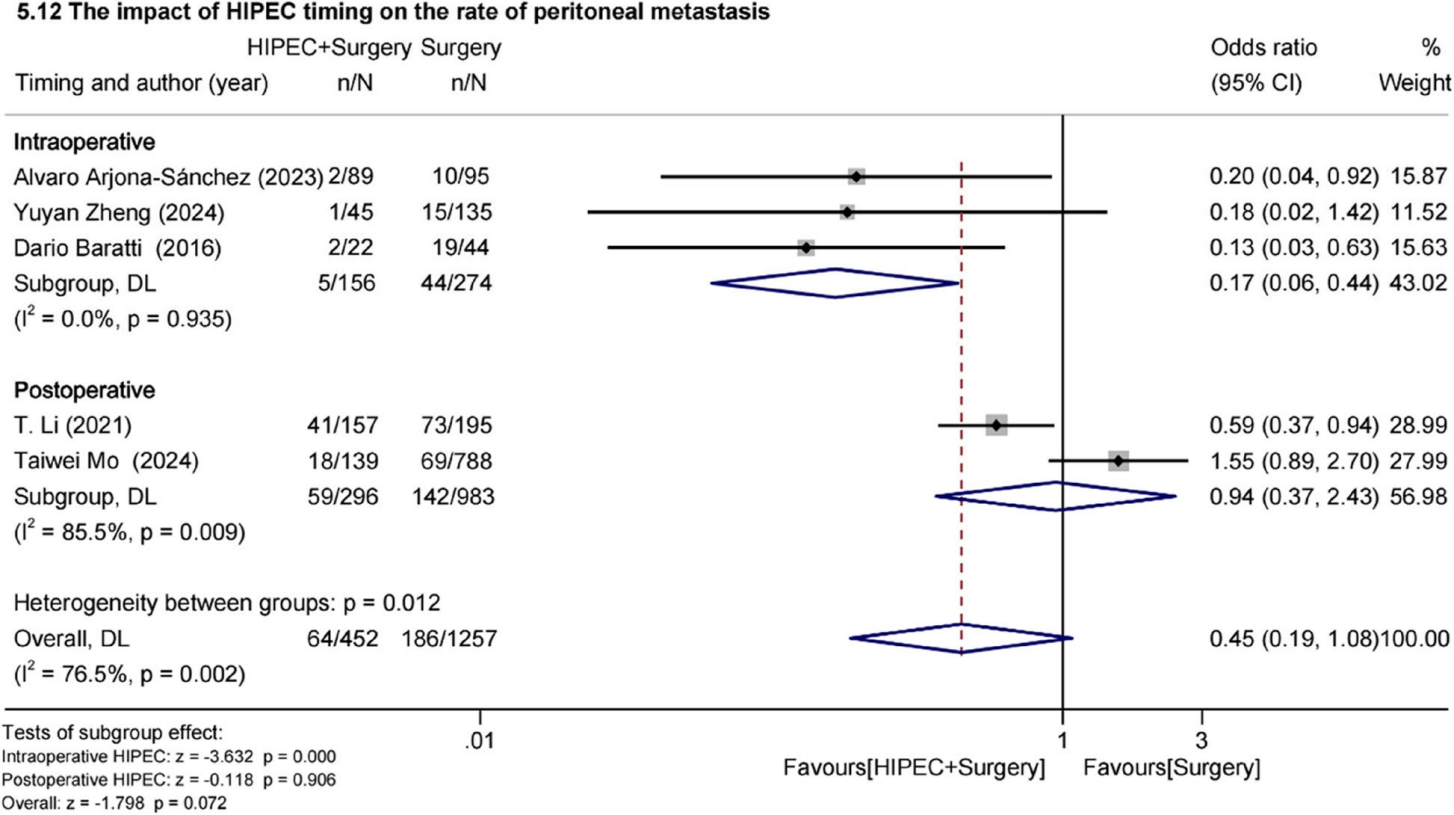
Effect of HIPEC timing on peritoneal metastasis rate

We conducted exploratory subgroup analysis based on the timing of HIPEC implementation. The results showed that the implementation of intraoperative HIPEC demonstrated a statistically significant reduction in PM rate (OR = 0.17, 95%Cl: 0.06–0.44, z=−3.632, *p* = 0.000 < 0.05). However, postoperative HIPEC showed no statistically significant difference (OR = 0.94, 95%Cl: 0.37–2.43, z=−0.118, *p* = 0.906 > 0.05) (Fig.7).

#### Summary of evidence

**Fig. 8:**
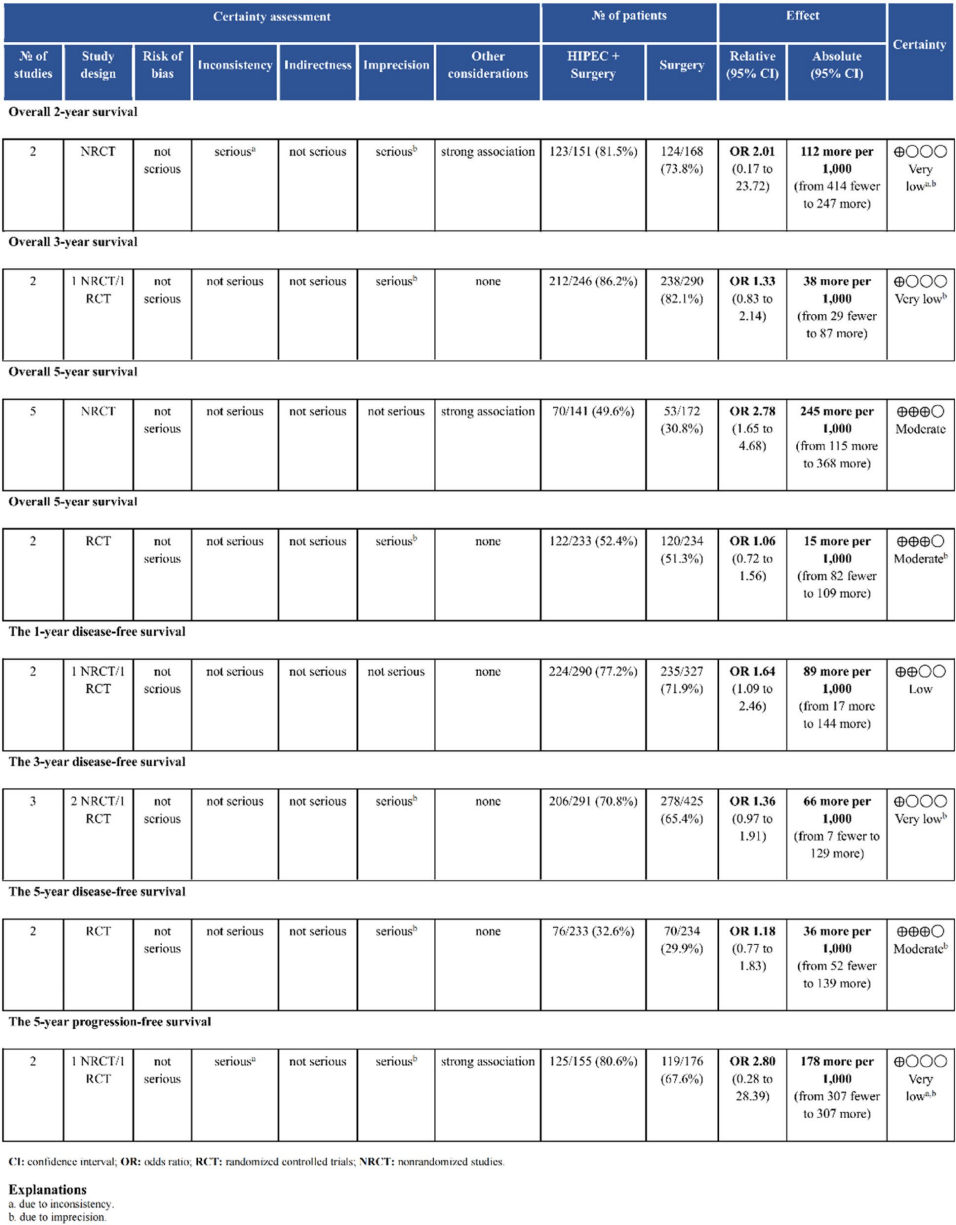
GRADE rating for quality of evidence about survival outcome

**Fig. 9:**
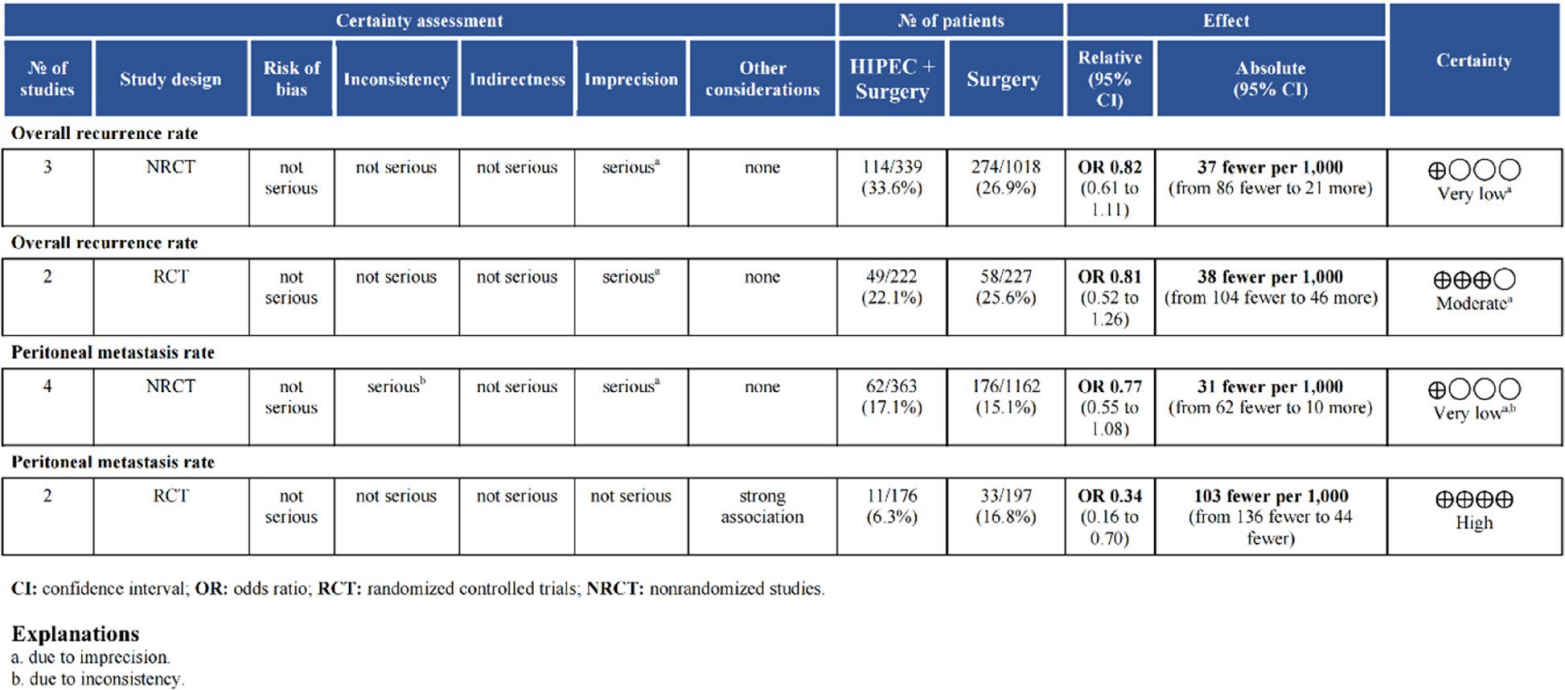
GRADE rating for quality of evidence about overall recurrence rate and peritoneal metastasis rate

We assessed the quality of evidence for both primary and secondary outcomes in the meta-analysis. For survival outcome, the quality of evidence ranged from very low to moderate (Fig.8). Both NRCTs and RCTs provided moderate-quality evidence for overall 5-year survival. For the outcomes of ORR and PM rate, the evidence quality from NRCTs was very low. The RCT-derived evidence was of moderate quality for ORR and high quality for PM rate (Fig. 9). Regarding complications, high-quality evidence was observed only for thrombopenia and neutropenia, while evidence for other complications was of very low quality (Appendix Fig. 8) .

## Discussion

In this study, we conducted a comprehensive meta-analysis of survival, recurrence, and complications of HIPEC in advanced CRC. The results suggest that HIPEC may be associated with improved 1-year DFS and 5-year OS. Additionally, HIPEC appears to lower the incidence of PM, although no clear benefit was observed in terms of reducing the ORR. In locally advanced high-risk CRC without previous PM, the use of HIPEC may serve to prevent PM. Intraoperative HIPEC, as opposed to postoperative use, may be related to a lower PM rate. Regarding adverse effects, HIPEC is generally considered safe, but may increase the risk of neutropenia and thrombocytopenia. The quality of evidence varied across different outcomes and should be interpreted with caution.

CRS-HIPEC is considered a beneficial option for patients with peritoneal carcinomatosis secondary to gastrointestinal or ovarian malignancies in the absence of extra-abdominal spread [36–38]. However, the role of HIPEC in advanced CRC is inconclusive [8]. The results of our analysis showed that HIPEC on top of curative surgery significantly improved 1-year DFS and 5-year OS in patients with advanced CRC. Similarly, a recent meta-analysis found that HIPEC was significantly superior to standard therapy in the treatment of advanced GC in terms of 3-year OS (OR = 1.89, 95%CI: 1.17–3.05) and 5-year OS (OR 1.87, 95%CI: 1.29–2.71) [39]. The ability of HIPEC to improve survival outcomes in advanced cancers has been linked to mechanisms such as direct cytotoxicity to tumor cells, synergistic effects with some chemotherapeutic agents, enhanced tissue penetration of the drugs, and increased infiltration of tumor immune cells [40].

However, in cases of extensive peritoneal involvement, the use of HIPEC may not be beneficial. Baratti D et al. found that decreased survival rate in CRC-PM was significantly associated with extraperitoneal disease and peritoneal residual lesions > 2.5 mm[41]. In addition, HIPEC is more likely to prolong survival outcomes with limited lymph node involvement. M Ikeguchi et al. found that only GC patients with 1–9 lymph node metastases had a potential 5-year survival benefit compared to those with 10 or more metastases [42]. In a multi-institutional analysis of two phase II clinical trials, a negative correlation was observed between the number of pathologically positive lymph nodes and OS in GC-PM treated with CRS-HIPEC (HR = 1.105, 95%CI: 1.006–1.213) [43]. Therefore, HIPEC may have the greatest survival benefit in patients with advanced CRC who have positive cytology, limited peritoneal involvement, and a limited number of metastatic lymph nodes.

In terms of reducing metastasis and recurrence, our study showed that HIPEC reduced the ORR but not significantly. HIPEC significantly reduced the peritoneal recurrence rate in patients with advanced CRC. A number of studies have also confirmed that HIPEC reduces peritoneal metastasis and recurrence of gastrointestinal tumor. A retrospective clinical study showed that HIPEC reduced the overall recurrence/metastasis rate, especially the PM rate, in GC patients after D2 resection [44]. Similar results were likewise reported in a meta-analysis conducted by Maitreyi Patel et al. that HIPEC reduced the peritoneal recurrence rate in patients with advanced GC (OR = 0.22, 95%CI: 0.11–0.47) [39]. The lower rate of peritoneal recurrence with HIPEC may be associated with improved OS[45].

The majority of CRC patients will develop metastases and more than half will do so even if diagnosed early [46]. Peritoneal metastases imply poor prognosis and short life expectancy. Therefore, identifying effective strategies to prevent PM is essential for improving patient survival and quality of life. Locally advanced disease (stage T4) is an important risk factor for heterochronous PM in CRC [47, 48]. In the included studies without PM, most CRC cases were stage T4. And the risk of developing PM was high in all cases. We performed subgroup analyses based on the occurrence or non-occurrence of PM. The results showed that for advanced high-risk CRC without PM, HIPEC significantly reduced the rate of PM and had a preventive effect. Similarly, a meta-analysis found that prophylactic HIPEC treatment significantly reduced the incidence of PM in CRC patients at high risk (RR: 0.41; 95% CI: 0.21–0.83) [49]. This meta-analysis and our study both involved the assessment of HIPEC, but differed in terms of inclusion criteria and statistical methods. Our study focuses on different patients those with CRC-PM and locally advanced CRC (stage T3-4) at high risk of PM rather than patients merely at risk of PM. We also applied stricter inclusion criteria, required consistent potentially curative surgery across groups, and conducted separate analyses by study design. In addition, intraoperative HIPEC showed a lower PM rate compared with postoperative HIPEC, possibly due to more thorough drug perfusion during surgery.

Studies have shown that in most peritoneal metastases, cytokines and chemokines such as TGF-β and thrombospondin-1 mediate the peritoneal immune microenvironment to exhibit an immunosuppressive phenotype, thereby promoting tumor invasion [50]. Hyperthermia has been demonstrated to modulate the infiltration of immune cells such as natural killer cells, CD4(+) and CD8(+) T cells thereby enhancing immunity and inhibiting cancer cell metastasis [51]. This may be one of the mechanisms by which HIPEC can prevent and treat peritoneal metastasis and recurrence.

However, the potential risks associated with HIPEC may limit its benefits. The most common complications of HIPEC are anastomotic leakage, intestinal perforation, hematological complications, and infections [52]. We evaluated adverse reactions such as diarrhea, nausea or vomiting, anastomotic leakage, anastomotic hemorrhage, infection (wound, Abdominal, Pulmonary), anemia. The analysis revealed no statistically significant difference in these complications. In one meta-analysis of 617 patients with ovarian cancer, HIPEC did not show significant toxicity [53]. However, our results additionally showed that HIPEC significantly increased the risk of neutropenia and thrombopenia. The occurrence of these adverse effects may be related to the fact that HIPEC can induce a heat shock response, increase systemic uptake of chemotherapy, and cause peritoneal surface burns [40]. Studies have shown that the experience of the clinical center performing HIPEC may be associated with postoperative morbidity and mortality from HIPEC [54]. Therefore, the safety of HIPEC for clinical treatment can be affirmed in experienced centers. To improve the safety of HIPEC, researchers are developing drug delivery systems that maximize intraperitoneal drug concentration and simultaneously reduce systemic toxicity. Examples include hydrogels, implants, nanoparticles, and hybrid systems [55].Currently, there is no consensus on the chemotherapeutic agents, perfusion duration, or optimal temperature used in most HIPEC regimens [8, 56]. Differences in drugs, duration, and temperature can affect the actual efficacy and safety of HIPEC. Given the constrained quantity of researches available for review, it was not feasible to conduct an assessment of these effects. Of the studies we included, most preferred mitomycin-C or oxaliplatin alone or in combination, or the addition of other drugs such as cisplatin, lobaplatin, raltitrexed, etc. The HIPEC procedure was conducted within a temperature range of 42 to 43.5℃, with a duration that varied from 30 to 90 min. Moreover, personalized HIPEC regimens may enhance therapeutic outcomes and minimize adverse effects in advanced CRC. Patient-derived organoid technology shows great potential in this regard, providing more precise drug therapy by constructing similar specific organs or originating tumors [57, 58].

Recent evidence has reshaped the understanding of HIPEC in colorectal peritoneal metastases. The PRODIGE 7 trial found no survival advantage for high-dose, short-duration oxaliplatin HIPEC, suggesting limited benefit and higher morbidity [21]. In contrast, earlier studies such as the Dutch trial reported improved outcomes with mitomycin-C-based protocols [59]. The COLOPEC trial, evaluating adjuvant HIPEC after resection of high-risk colon cancer, also failed to show benefit, underscoring the need for regimen optimization [23]. Current consensus favors MMC-based or multi-agent HIPEC regimens, emphasizing longer perfusion times and individualized protocols guided by ongoing clinical and preclinical research.

The current meta-analysis also has its limitations. Firstly, we included 10 cohort studies, which may have skewed the results. In response, we conducted subgroup analyses to show the results of RCTs and NRCTs separately. Secondly, varying degrees of heterogeneity were observed in the result analysis. We summarized the heterogeneity and the effect model used for each pooled outcome (Appendix Table 2). Because several included studies did not report whether patients received neoadjuvant therapy, this factor could not be controlled for and represents a potential source of heterogeneity. Thirdly, more than half of the included studies were conducted in China, while relatively few originated from Europe or other regions. Patient selection criteria, surgical expertise, and perioperative management differ across regions. Therefore, the geographical concentration of studies may limit the external validity of the findings to American and European populations. Further multicenter RCTs from diverse geographical settings are warranted. Fourthly, due to the constrained quantity of researches in our analysis, we were restrained from delving into the effects of variations in tumor pathology, staging, and HIPEC protocols on the ultimate efficacy and safety outcomes. We are also unable to conduct publication bias and sensitivity analysis. The results obtained from this study should be treated with caution. Moreover, because peritoneal cancer index values in our included studies were heterogeneous, we were also unable to analyze the impact of differences in peritoneal cancer index on HIPEC. Furthermore, most included studies did not report postoperative complications using standardized grading systems (e.g., CTCAE, Clavien–Dindo classification), which precluded unified comparison of complication severity.

## Conclusion

Our findings indicate a potential benefit of HIPEC in improving survival and reducing peritoneal recurrence in advanced CRC, but attention should be paid to the harmful propensity for complications. In CRC at high risk of PM, HIPEC may have a role in preventing PM. Given the small number of RCTs and potential publication bias, more large-scale, high-quality, multicenter RCTs are needed to further explore the role of HIPEC.

## Supplementary Information

Supplementary Material 1.

Supplementary Material 2.

Supplementary Material 3.

## Data Availability

Not applicable.

## Abbreviations

CI: Confidence intervals
CRC: Colorectal cancer
CRS: Cytoreductive surgery
DFS: Disease-free survival
GC: Gastric cancer
HIPEC: Hyperthermic intraperitoneal chemotherapy
NOS: the Newcastle-Ottawa Scale
OR: Odds ratio
ORR: Overall recurrence rate
OS: Overall survival
PFS: Progression-free survival
PICOS: Population, intervention, comparison, outcomes, and study design
PM: Peritoneal metastases
RCT: Randomized controlled trials
