# Supplementary material for: Effect of hyperthermic intraperitoneal chemotherapy on patients with advanced colorectal cancer: a systematic review and meta-analysis
Source: World J Surg Oncol. 2026 Jan 29;24:117. doi: 10.1186/s12957-025-04165-7 (PMC12980965; doi:10.1186/s12957-025-04165-7)
Supplement: Supplementary file 1 — Supplementary Material 1. [file 12957_2025_4165_MOESM1_ESM.pdf]

Effect of hyperthermic intraperitoneal chemotherapy on patients with advanced colorectal cancer: a systematic review and meta-analysis

Hyperthermic intraperitoneal chemotherapy (HIPEC)

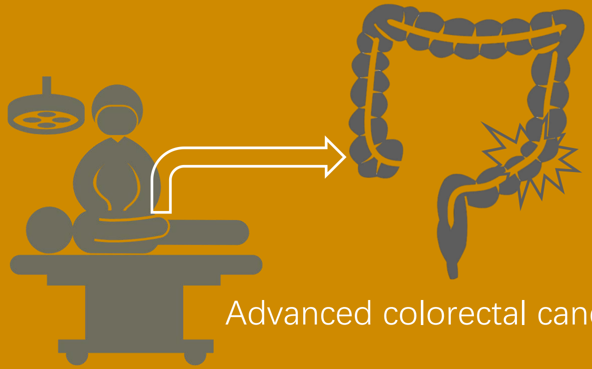

Advanced colorectal cancer (CRC)

- 10 high-quality cohort studies
- 4 randomized controlled trial
- n=2,851 patients

The aim is to comprehensively evaluate the standalone effect of HIPEC in advanced CRC.

1. In terms of survival

HIPEC significantly improved

1-year DFS OR =1.64, 95%CI: 1.09-2.46

5-year OS OR=1.49, 95%CI: 1.10-2.03

2. Regarding recurrence

- **Peritoneal metastasis (PM) and recurrence**

HIPEC significantly reduced **the overall PM rate**

OR=0.66, 95%CI: 0.49-0.90

**OR=0.13, 95%CI: 0.03-0.63**	**OR=0.71, 95%CI: 0.52-0.97**
**Therapeutic HIPEC**	**Preventive HIPEC**
Reduced peritoneal recurrence for previous PM	Reduced PM rate without previous PM

- **No significant effect of HIPEC on overall recurrence rate**

3. In complications

Neutropenia OR=3.21, 95%CI: 1.74-5.90

Thrombopenia OR=5.77, 95%CI: 1.65-20.09

Conclusion

1. The use of HIPEC in the treatment of advanced CRC may result in improved survival rates and a reduction in peritoneal recurrence, but complications should be noted.

2. In patients with advanced CRC who have not developed PM, the use of HIPEC may serve to prevent PM.

Visual Abstract
