# Supplementary material for: Effect of hyperthermic intraperitoneal chemotherapy on patients with advanced colorectal cancer: a systematic review and meta-analysis
Source: World J Surg Oncol. 2026 Jan 29;24:117. doi: 10.1186/s12957-025-04165-7 (PMC12980965; doi:10.1186/s12957-025-04165-7)
Supplement: Supplementary file 2 — Supplementary Material 2. [file 12957_2025_4165_MOESM2_ESM.docx]

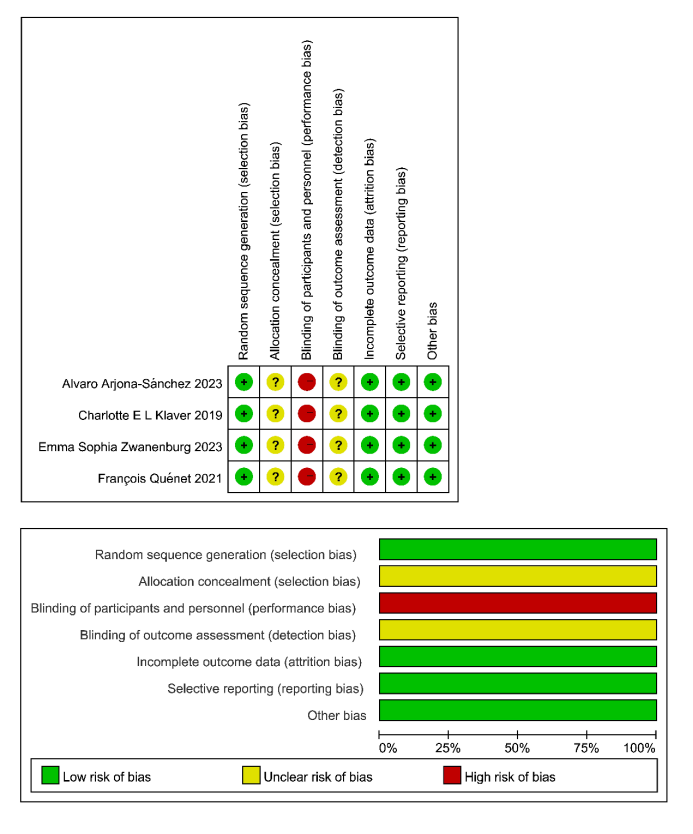


Appendix Figure 1. Risk of bias assessment for randomized controlled trials.


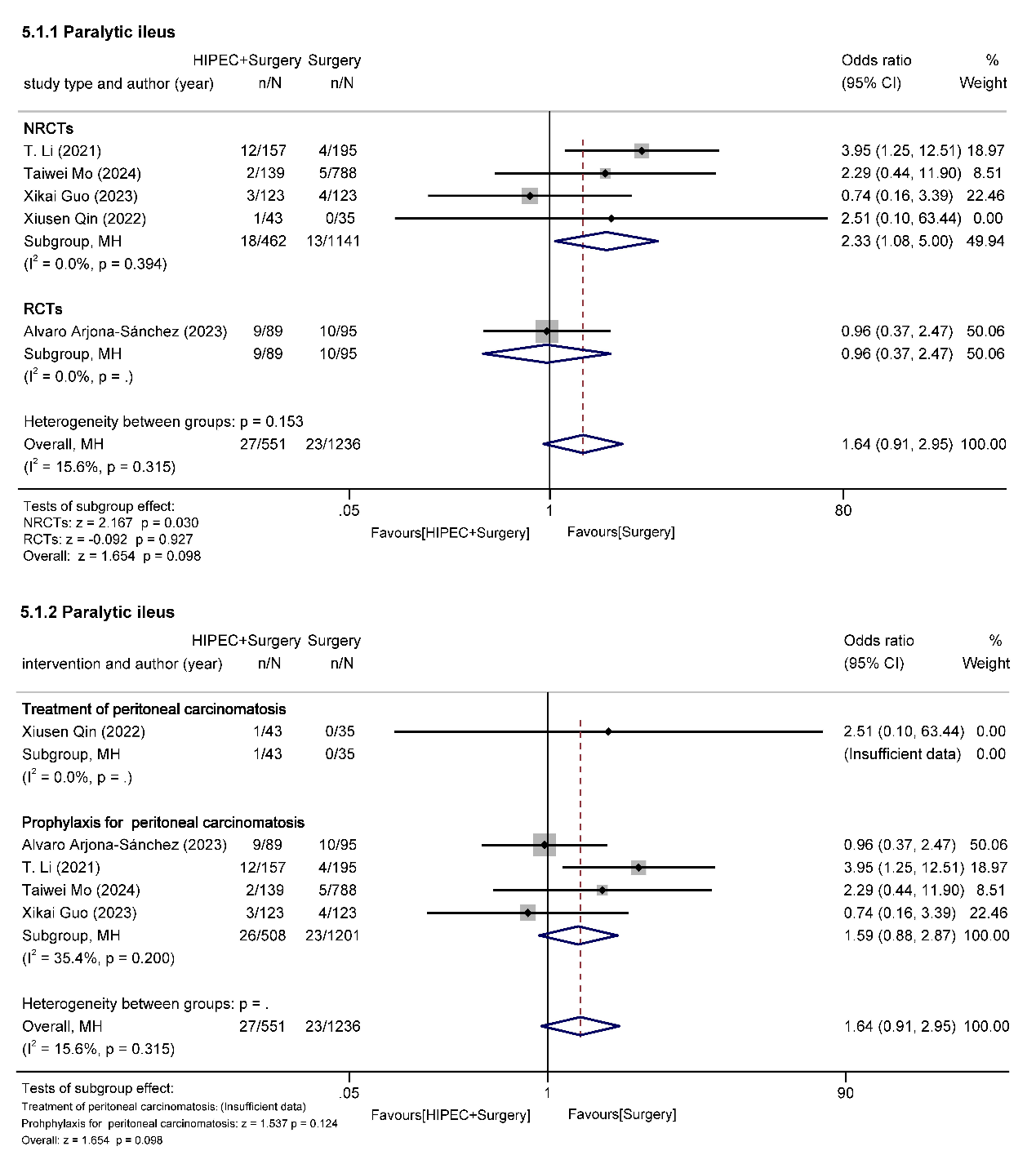


Appendix Figure 2. Effect of HIPEC on paralytic ileus.


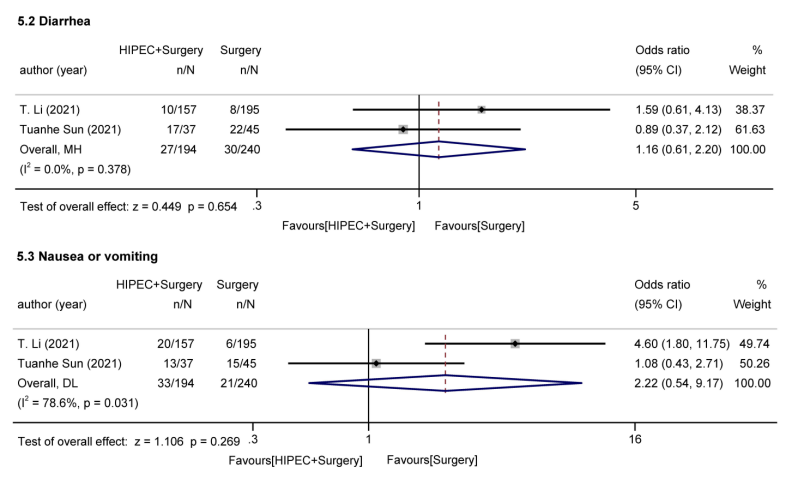


Appendix Figure 3. Effect of HIPEC on diarrhea and nausea or vomiting.


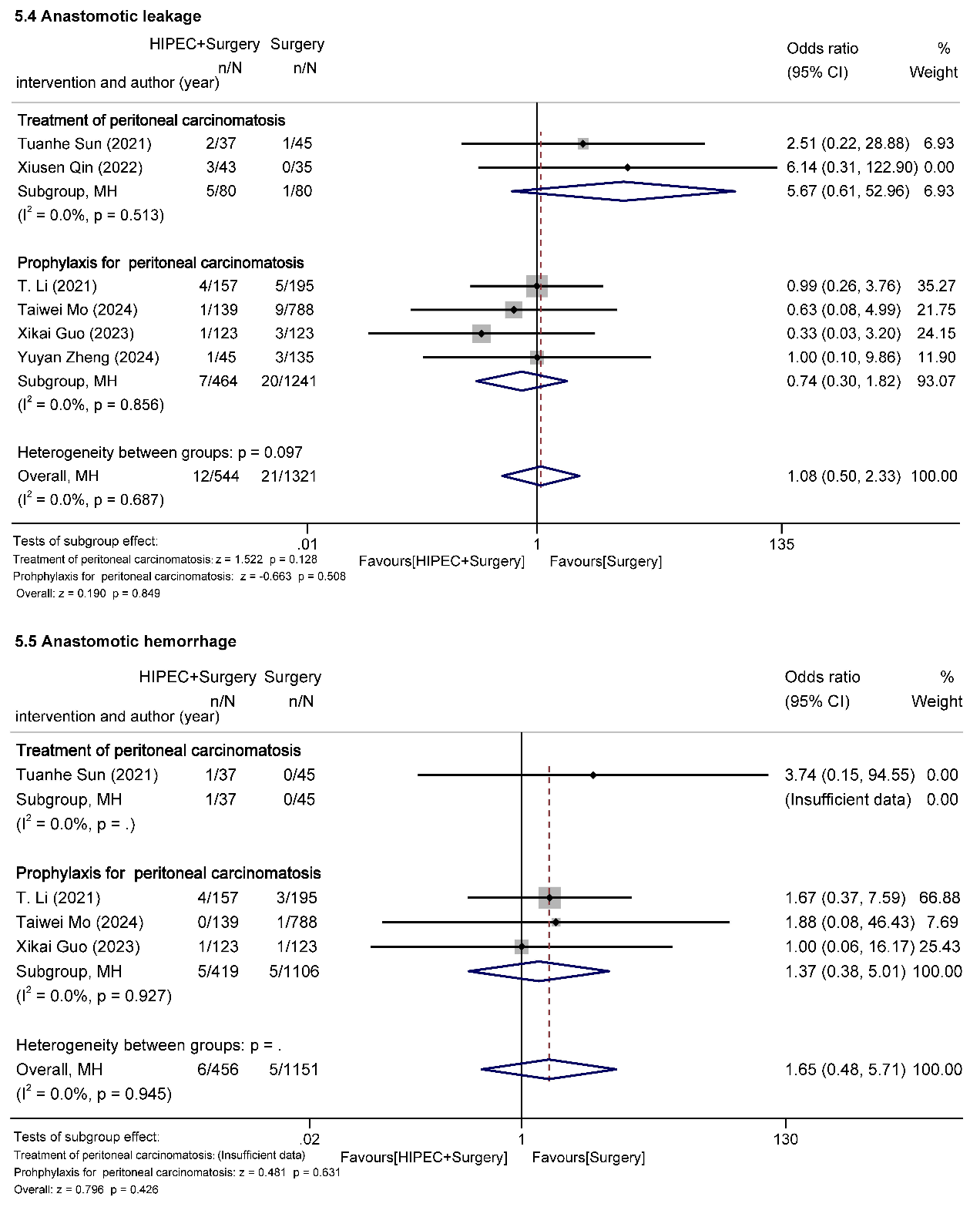


Appendix Figure 4. Effect of HIPEC on anastomotic leakage and anastomotic hemorrhage.


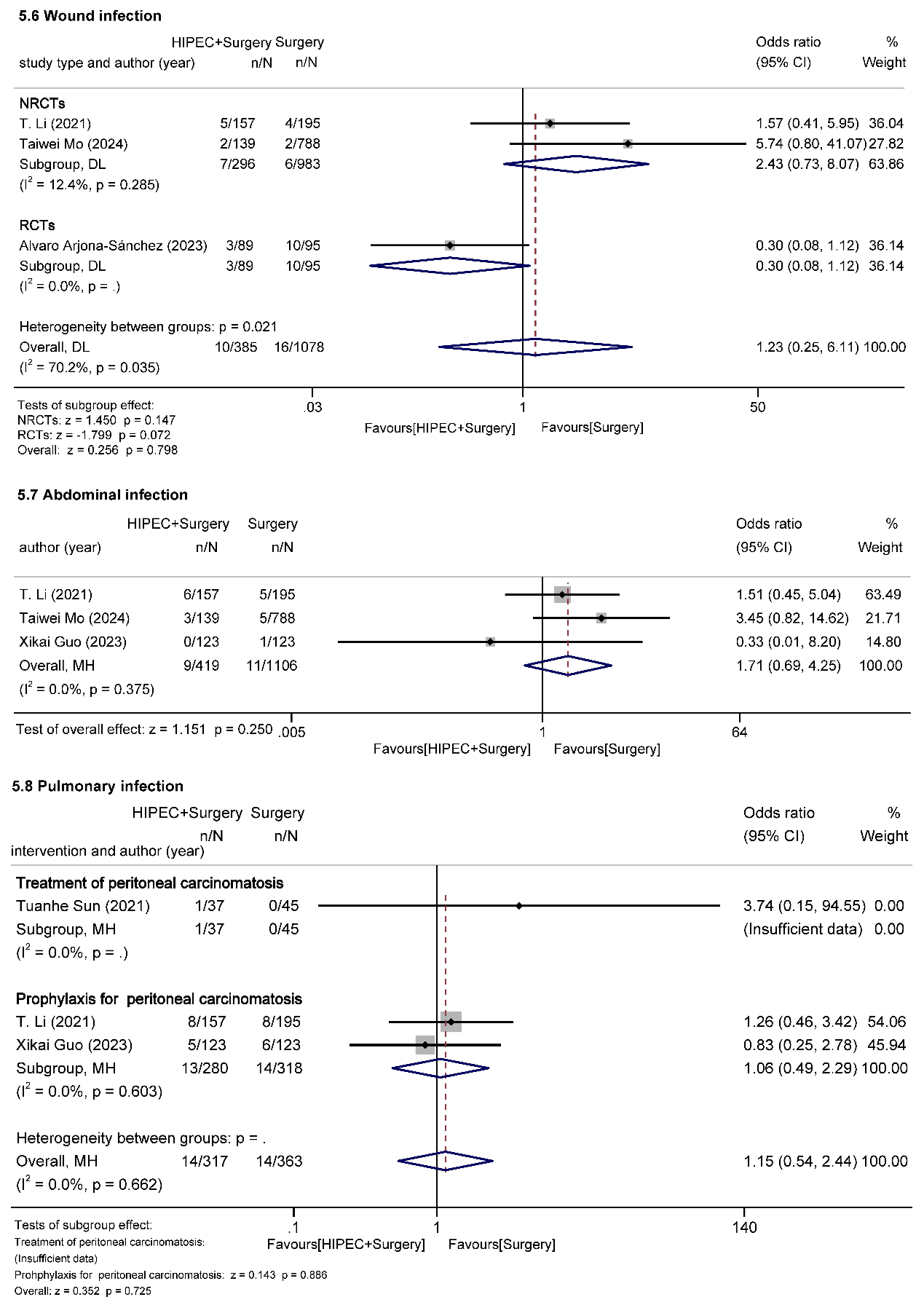


Appendix Figure 5. Effect of HIPEC on wound, abdominal and pulmonary infection.


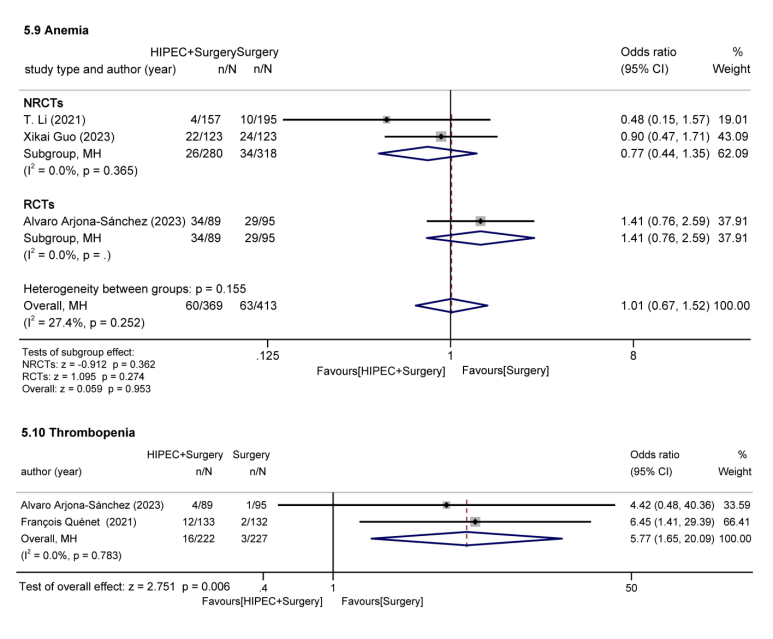


Appendix Figure 6. Effect of HIPEC on anemia and thrombopenia.


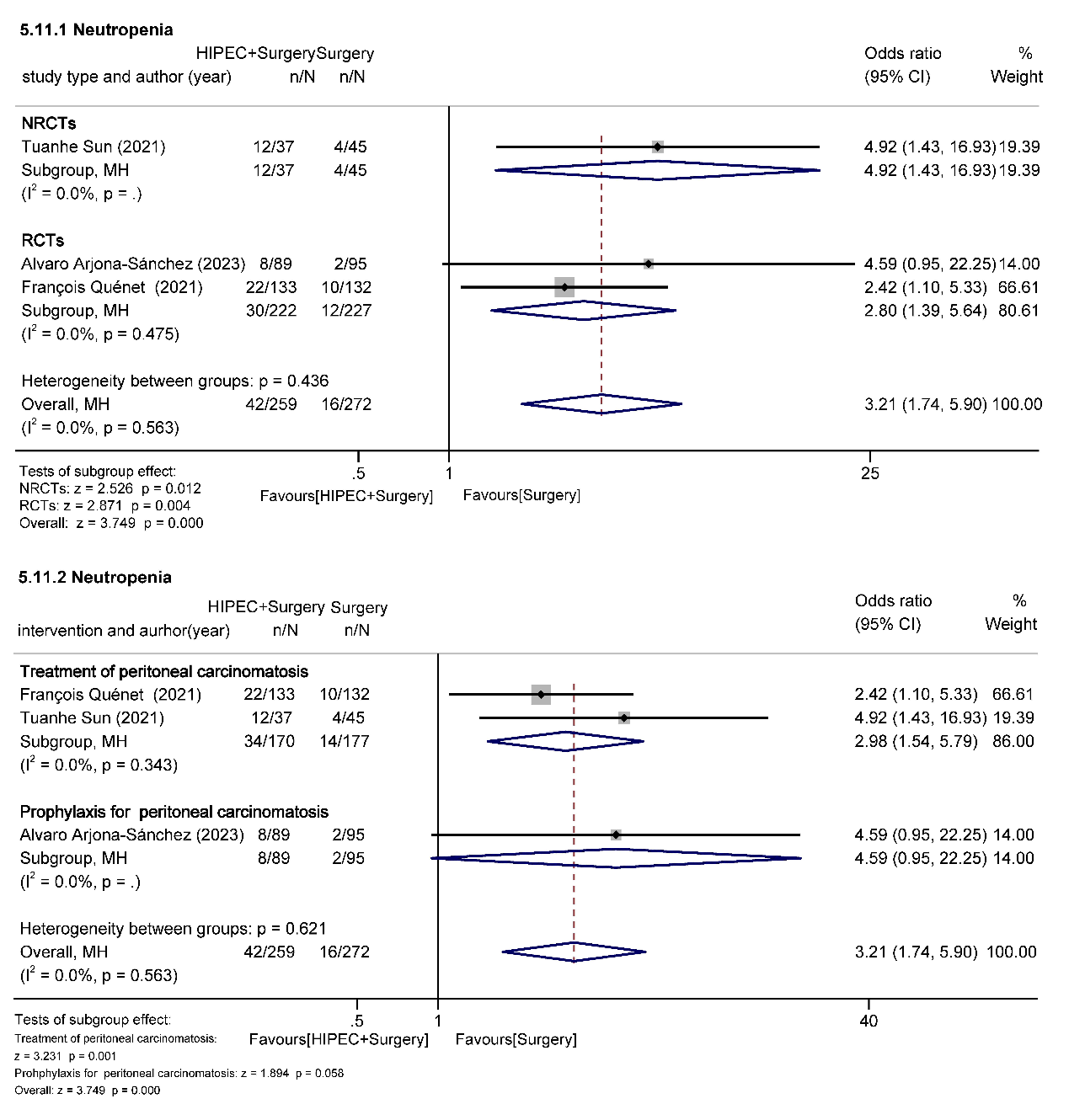


Appendix Figure 7. Effect of HIPEC on neutropenia.


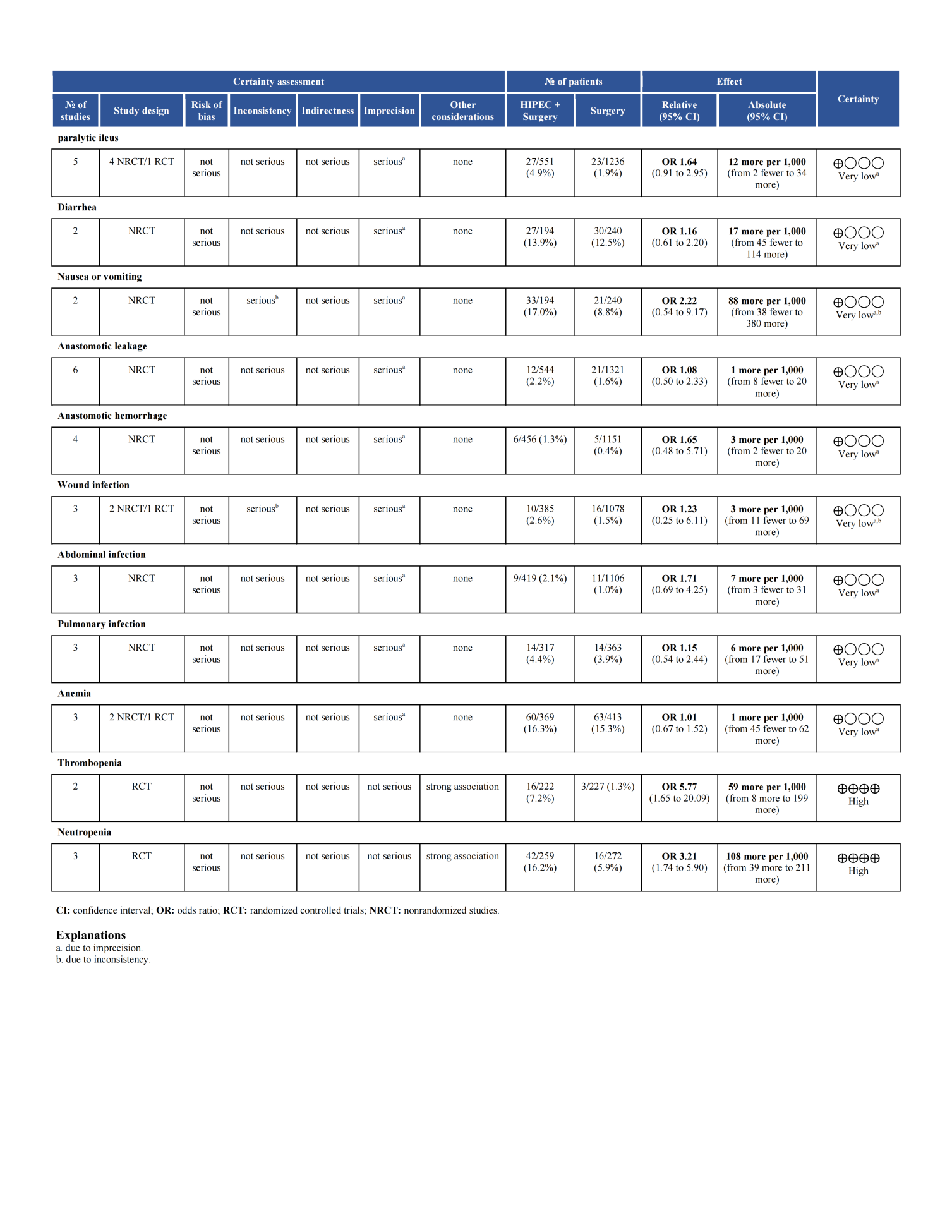


Appendix Figure 8. GRADE rating for quality of evidence about complications.

Appendix Table 1. Relevant medical subject headings in the search strategy.

| 1 | Colorectal Neoplasms |
| --- | --- |
| 2 | Colonic Neoplasms |
| 3 | Rectal Neoplasms |
| 4 | Hyperthermic Intraperitoneal Chemotherapy |

Appendix Table 2. Summary of pooled outcomes, heterogeneity, and effect models

| **Outcome** | **No. of studies** | **OR (95% CI)** | **I² (%)** | **p**  **(heterogeneity)** | **Model used** |
| --- | --- | --- | --- | --- | --- |
| Overall 2-year survival | 2 | 2.01 (0.17-23.72) | 92.9 | 0.000 | random effects model |
| Overall 3-year survival | 2 | 1.33 (0.83-2.14) | 0.0 | 0.479 | fixed effect model |
| Overall 5-year survival | 6 | 1.49 (1.10-2.03) | 51.6 | 0.067 | fixed effect model |
| The 1-year disease-free survival | 2 | 1.64 (1.09-2.46) | 0.0 | 0.948 | fixed effect model |
| The 3-year disease-free survival | 3 | 1.36 (0.97-1.91) | 0.0 | 0.448 | fixed effect model |
| The 5-year disease-free survival | 2 | 1.18 (0.77-1.83) | 0.0 | 0.969 | fixed effect model |
| Overall recurrence rate | 5 | 0.82 (0.64-1.05) | 0.0 | 0.464 | fixed effect model |
| Peritoneal metastasis rate | 6 | 0.66 (0.49-0.90) | 73.3 | 0.002 | random effects model |
| Paralytic ileus | 5 | 1.64 (0.91-2.95) | 15.6 | 0.315 | fixed effect model |
| Diarrhea | 2 | 1.16 (0.61-2.20) | 0.0 | 0.378 | fixed effect model |
| Nausea or vomiting | 2 | 2.22 (0.54-9.17) | 78.6 | 0.031 | random effects model |
| Anastomotic leakage | 6 | 1.08 (0.50-2.33) | 0.0 | 0.687 | fixed effect model |
| Anastomotic hemorrhage | 4 | 1.65 (0.48-5.71) | 0.0 | 0.945 | fixed effect model |
| Wound infection | 3 | 1.23 (0.25,6.11) | 70.2 | 0.035 | random effects model |
| Abdominal infection | 3 | 1.71 (0.69,4.25) | 0.0 | 0.375 | fixed effect model |
| Pulmonary infection | 3 | 1.15 (0.54,2.44) | 0.0 | 0.662 | fixed effect model |
| Anemia | 3 | 1.01 (0.67,1.52) | 27.4 | 0.252 | fixed effect model |
| Thrombopenia | 2 | 5.77 (1.65,20.09) | 0.0 | 0.783 | fixed effect model |
| Neutropenia | 3 | 3.21 (1.74,5.90) | 0.0 | 0.563 | fixed effect model |

Appendix Table 3. Characteristics of the studies included.

| Authors (Year) | Peritoneal Cancer Index | Extent of cytoreduction | Timing of HIPEC  (intraoperative vs. postoperative) |
| --- | --- | --- | --- |
| Baratti et al. (2016) | PCI ≈ 0–3 | Radical R0 resection/CCR-0 | intraoperative |
| Zheng et al. (2024) | PCI = 0 | Radical R0 resection | intraoperative |
| Arjona-Sánchez et al. (2023) | PCI = 0 | Radical R0 resection | intraoperative |
| Li et al. (2021) | PCI = 0 | Radical R0 resection | postoperative |
| Mo et al. (2024) | PCI = 0 | Radical R0 resection | postoperative |
| Klaver et al. (2019) | The PCI score was not systematically determined in some patients. | No initial comprehensive cytoreductive surgery was performed. | intraoperative or postoperative |
