# Supplementary material for: Effect of hyperthermic intraperitoneal chemotherapy on patients with advanced colorectal cancer: a systematic review and meta-analysis
Source: World J Surg Oncol. 2026 Jan 29;24:117. doi: 10.1186/s12957-025-04165-7 (PMC12980965; doi:10.1186/s12957-025-04165-7)
Supplement: Supplementary file 3 — Supplementary Material 3. [file 12957_2025_4165_MOESM3_ESM.docx]

**Retrieval record**

**Table 1: PubMed 912 results (12 April, 2024)**

| **Search** | **Query** | **Results** |
| --- | --- | --- |
| #3 | #1 AND #2 | 912 |
| #2 | (("Hyperthermic Intraperitoneal Chemotherapy"[Mesh]) OR (((((((((Chemotherapy, Hyperthermic Intraperitoneal[Title/Abstract]) OR (Intraperitoneal Chemotherapy, Hyperthermic[Title/Abstract])) OR (HIPEC[Title/Abstract])) OR (Hot Chemotherapy[Title/Abstract])) OR (Chemotherapy, Hot[Title/Abstract])) OR (Intraperitoneal Hyperthermic Chemotherapy[Title/Abstract])) OR (Chemotherapy, Intraperitoneal Hyperthermic[Title/Abstract])) OR (Hyperthermic Chemotherapy, Intraperitoneal[Title/Abstract])) OR (Intraperitoneal Hyperthermic Chemotherapies[Title/Abstract]))) | 5,030 |
| #1 | ((((((((((((((((((((((((((((((((((((((((((((((((((((((((Colorectal Neoplasms[Title/Abstract]) OR (Colorectal Neoplasm[Title/Abstract])) OR (Neoplasm, Colorectal[Title/Abstract])) OR (Neoplasms, Colorectal[Title/Abstract])) OR (Colorectal Tumors[Title/Abstract])) OR (Colorectal Tumor[Title/Abstract])) OR (Tumor, Colorectal[Title/Abstract])) OR (Tumors, Colorectal[Title/Abstract])) OR (Colorectal Cancer[Title/Abstract])) OR (Cancer, Colorectal[Title/Abstract])) OR (Cancers, Colorectal[Title/Abstract])) OR (Colorectal Cancers[Title/Abstract])) OR (Colorectal Carcinoma[Title/Abstract])) OR (Carcinomas, Colorectal[Title/Abstract])) OR (Carcinoma, Colorectal[Title/Abstract])) OR (Colorectal Carcinomas[Title/Abstract])) OR (Colonic Neoplasms[Title/Abstract])) OR (Colonic Neoplasm[Title/Abstract])) OR (Neoplasm, Colonic[Title/Abstract])) OR (Neoplasms, Colonic[Title/Abstract])) OR (Colon Neoplasms[Title/Abstract])) OR (Colon Neoplasm[Title/Abstract])) OR (Neoplasm, Colon[Title/Abstract])) OR (Neoplasms, Colon[Title/Abstract])) OR (Cancer of Colon[Title/Abstract])) OR (Colon Cancers[Title/Abstract])) OR (Colon Cancer[Title/Abstract])) OR (Cancer, Colon[Title/Abstract])) OR (Cancers, Colon[Title/Abstract])) OR (Cancer of the Colon[Title/Abstract])) OR (Colonic Cancer[Title/Abstract])) OR (Cancer, Colonic[Title/Abstract])) OR (Cancers, Colonic[Title/Abstract])) OR (Colonic Cancers[Title/Abstract])) OR (Colon Adenocarcinoma[Title/Abstract])) OR (Adenocarcinoma, Colon[Title/Abstract])) OR (Adenocarcinomas, Colon[Title/Abstract])) OR (Colon Adenocarcinomas[Title/Abstract])) OR (Rectal Neoplasms[Title/Abstract])) OR (Neoplasm, Rectal[Title/Abstract])) OR (Rectal Neoplasm[Title/Abstract])) OR (Rectum Neoplasms[Title/Abstract])) OR (Neoplasm, Rectum[Title/Abstract])) OR (Rectum Neoplasm[Title/Abstract])) OR (Rectal Tumors[Title/Abstract])) OR (Rectal Tumor[Title/Abstract])) OR (Tumor, Rectal[Title/Abstract])) OR (Neoplasms, Rectal[Title/Abstract])) OR (Cancer of Rectum[Title/Abstract])) OR (Rectum Cancers[Title/Abstract])) OR (Rectal Cancer[Title/Abstract])) OR (Cancer, Rectal[Title/Abstract])) OR (Rectal Cancers[Title/Abstract])) OR (Rectum Cancer[Title/Abstract])) OR (Cancer, Rectum[Title/Abstract])) OR (Cancer of the Rectum[Title/Abstract])) | 241,081 |

**Table 2: Embase 2286 results (12 April, 2024)**

| **Search** | **Query** | **Results** |
| --- | --- | --- |
| #11 | #7 AND #10 | 2286 |
| #10 | #8 OR #9 | 7173 |
| #9 | 'chemotherapy, hyperthermic intraperitoneal':ab,ti OR 'intraperitoneal chemotherapy, hyperthermic':ab,ti OR 'hipec':ab,ti OR 'hot chemotherapy':ab,ti OR 'chemotherapy, hot':ab,ti OR 'intraperitoneal hyperthermic chemotherapy':ab,ti OR 'chemotherapy, intraperitoneal hyperthermic':ab,ti OR 'hyperthermic chemotherapy, intraperitoneal':ab,ti OR 'intraperitoneal hyperthermic chemotherapies':ab,ti | 5814 |
| #8 | 'hyperthermic intraperitoneal chemotherapy'/exp | 5342 |
| #7 | #1 OR #2 OR #3 OR #4 OR #5 OR #6 | 523598 |
| #6 | 'neoplasm, rectal':ab,ti OR 'rectal neoplasm':ab,ti OR 'rectum neoplasms':ab,ti OR 'neoplasm, rectum':ab,ti OR 'rectum neoplasm':ab,ti OR 'rectal tumors':ab,ti OR 'rectal tumor':ab,ti OR 'tumor, rectal':ab,ti OR 'neoplasms, rectal':ab,ti OR 'cancer of rectum':ab,ti OR 'rectum cancers':ab,ti OR 'rectal cancer':ab,ti OR 'cancer, rectal':ab,ti OR 'rectal cancers':ab,ti OR 'rectum cancer':ab,ti OR 'cancer, rectum':ab,ti OR 'cancer of the rectum':ab,ti OR 'rectal neoplasms':ab,ti | 53113 |
| #5 | 'rectum tumor'/exp | 86202 |
| #4 | 'colonic neoplasm':ab,ti OR 'neoplasm, colonic':ab,ti OR 'neoplasms, colonic':ab,ti OR 'colon neoplasms':ab,ti OR 'colon neoplasm':ab,ti OR 'neoplasm, colon':ab,ti OR 'neoplasms, colon':ab,ti OR 'cancer of colon':ab,ti OR 'colon cancers':ab,ti OR 'cancers, colon':ab,ti OR 'colon cancer':ab,ti OR 'cancer, colon':ab,ti OR 'cancer of the colon':ab,ti OR 'colonic cancer':ab,ti OR 'cancer, colonic':ab,ti OR 'cancers, colonic':ab,ti OR 'colonic cancers':ab,ti OR 'colon adenocarcinoma':ab,ti OR 'adenocarcinoma, colon':ab,ti OR 'adenocarcinomas, colon':ab,ti OR 'colon adenocarcinomas':ab,ti OR 'colonic neoplasms':ab,ti | 98592 |
| #3 | 'colon tumor'/exp | 185056 |
| #2 | 'colorectal tumor'/exp | 490830 |
| #1 | 'colorectal neoplasm':ab,ti OR 'neoplasm, colorectal':ab,ti OR 'neoplasms, colorectal':ab,ti OR 'colorectal tumors':ab,ti OR 'colorectal neoplasms':ab,ti OR 'tumor, colorectal':ab,ti OR 'tumors, colorectal':ab,ti OR 'colorectal cancer':ab,ti OR 'cancer, colorectal':ab,ti OR 'cancers, colorectal':ab,ti OR 'colorectal cancers':ab,ti OR 'colorectal carcinoma':ab,ti OR 'carcinoma, colorectal':ab,ti OR 'carcinomas, colorectal':ab,ti OR 'colorectal carcinomas':ab,ti | 227836 |

**Table 3: Web of Science 1864 results (April 12, 2024)**

| **Search** | **Query** | **Results** |
| --- | --- | --- |
| #1 | ((((((((((((((((((((((((((((((((((((((((((((((((((((((((TS=(Colorectal Neoplasms)) OR TS=(Colorectal Neoplasm)) OR TS=(Neoplasm, Colorectal)) OR TS=(Neoplasms, Colorectal)) OR TS=(Colorectal Tumors)) OR TS=(Colorectal Tumor)) OR TS=(Tumor, Colorectal)) OR TS=(Tumors, Colorectal)) OR TS=(Colorectal Cancer)) OR TS=(Cancer, Colorectal)) OR TS=(Cancers, Colorectal)) OR TS=(Colorectal Cancers)) OR TS=(Colorectal Carcinoma)) OR TS=(Carcinoma, Colorectal)) OR TS=(Carcinomas, Colorectal)) OR TS=(Colorectal Carcinomas)) OR TS=(Colonic Neoplasms)) OR TS=(Colonic Neoplasm)) OR TS=(Neoplasm, Colonic)) OR TS=(Neoplasms, Colonic)) OR TS=(Colon Neoplasms)) OR TS=(Colon Neoplasm)) OR TS=(Neoplasm, Colon)) OR TS=(Neoplasms, Colon)) OR TS=(Cancer of Colon)) OR TS=(Colon Cancers)) OR TS=(Colon Cancer)) OR TS=(Cancer, Colon)) OR TS=(Cancers, Colon)) OR TS=(Cancer of the Colon)) OR TS=(Colonic Cancer)) OR TS=(Cancer, Colonic)) OR TS=(Cancers, Colonic)) OR TS=(Colonic Cancers)) OR TS=(Colon Adenocarcinoma)) OR TS=(Adenocarcinoma, Colon)) OR TS=(Adenocarcinomas, Colon)) OR TS=(Colon Adenocarcinomas)) OR TS=(Rectal Neoplasms)) OR TS=(Neoplasm, Rectal)) OR TS=(Rectal Neoplasm)) OR TS=(Rectum Neoplasms)) OR TS=(Neoplasm, Rectum))) OR TS=(Neoplasm, Rectum)) OR TS=(Rectal Tumors)) OR TS=(Rectal Tumor)) OR TS=(Tumor, Rectal)) OR TS=(Neoplasms, Rectal)) OR TS=(Cancer of Rectum)) OR TS=(Rectum Cancers)) OR TS=(Rectal Cancer)) OR TS=(Cancer, Rectal)) OR TS=(Rectal Cancers)) OR TS=(Rectum Cancer)) OR TS=(Cancer, Rectum)) OR TS=(Cancer of the Rectum) | 284603 |
| #2 | (((((((((TS=(Hyperthermic Intraperitoneal Chemotherapy)) OR TS=(Chemotherapy, Hyperthermic Intraperitoneal)) OR TS=(Intraperitoneal Chemotherapy, Hyperthermic)) OR TS=(HIPEC)) OR TS=(Hot Chemotherapy)) OR TS=(Chemotherapy, Hot)) OR TS=(Intraperitoneal Hyperthermic Chemotherapy)) OR TS=(Chemotherapy, Intraperitoneal Hyperthermic)) OR TS=(Hyperthermic Chemotherapy, Intraperitoneal)) OR TS=(Intraperitoneal Hyperthermic Chemotherapies) | 6036 |
| #3 | #2 AND #1 | 1864 |

**Table 4: Cochrane Library 228 results (April 12, 2024)**

| **Search** | **Query** | **Results** |
| --- | --- | --- |
| #1 | MeSH descriptor: [Colorectal Neoplasms] explode all trees | 12651 |
| #2 | (Colorectal Neoplasm):ti,ab,kw OR (Neoplasm, Colorectal):ti,ab,kw OR (Neoplasms, Colorectal):ti,ab,kw OR (Colorectal Tumors):ti,ab,kw OR (Colorectal Tumor):ti,ab,kw OR (Tumor, Colorectal):ti,ab,kw OR (Tumors, Colorectal):ti,ab,kw OR (Colorectal Cancer):ti,ab,kw OR (Cancer, Colorectal):ti,ab,kw OR (Cancers, Colorectal):ti,ab,kw OR (Colorectal Cancers):ti,ab,kw OR (Colorectal Carcinoma):ti,ab,kw OR (Carcinoma, Colorectal):ti,ab,kw OR (Carcinomas, Colorectal):ti,ab,kw OR (Colorectal Carcinomas):ti,ab,kw | 20522 |
| #3 | MeSH descriptor: [Colonic Neoplasms] explode all trees | 2546 |
| #4 | (Colonic Neoplasm):ti,ab,kw OR (Neoplasm, Colonic):ti,ab,kw OR (Neoplasms, Colonic):ti,ab,kw OR (Colon Neoplasms):ti,ab,kw OR (Colon Neoplasm):ti,ab,kw OR (Neoplasm, Colon):ti,ab,kw OR (Neoplasms, Colon):ti,ab,kw OR (Cancer of Colon):ti,ab,kw OR (Colon Cancers):ti,ab,kw OR (Colon Cancer):ti,ab,kw OR (Cancer, Colon):ti,ab,kw OR (Cancers, Colon):ti,ab,kw OR (Cancer of the Colon):ti,ab,kw OR (Colonic Cancer):ti,ab,kw OR (Cancer, Colonic):ti,ab,kw OR (Cancers, Colonic):ti,ab,kw OR (Colonic Cancers):ti,ab,kw OR (Colon Adenocarcinoma):ti,ab,kw OR (Adenocarcinoma, Colon):ti,ab,kw OR (Adenocarcinomas, Colon):ti,ab,kw OR (Colon Adenocarcinomas):ti,ab,kw | 9237 |
| #5 | MeSH descriptor: [Rectal Neoplasms] explode all trees | 2855 |
| #6 | (Neoplasm, Rectal):ti,ab,kw OR (Rectal Neoplasm):ti,ab,kw OR (Rectum Neoplasms):ti,ab,kw OR (Neoplasm, Rectum):ti,ab,kw OR (Rectum Neoplasm):ti,ab,kw OR (Rectal Tumors):ti,ab,kw OR (Rectal Tumor):ti,ab,kw OR (Tumor, Rectal):ti,ab,kw OR (Neoplasms, Rectal):ti,ab,kw OR (Cancer of Rectum):ti,ab,kw OR (Rectum Cancers):ti,ab,kw OR (Rectal Cancer):ti,ab,kw OR (Cancer, Rectal):ti,ab,kw OR (Rectal Cancers):ti,ab,kw OR (Rectum Cancer):ti,ab,kw OR (Cancer, Rectum):ti,ab,kw OR (Cancer of the Rectum):ti,ab,kw | 9142 |
| #7 | #1 OR #2 OR #3 OR #4 OR #5 OR #6 | 30459 |
| #8 | MeSH descriptor: [Hyperthermic Intraperitoneal Chemotherapy] explode all trees | 86 |
| #9 | (Chemotherapy, Hyperthermic Intraperitoneal):ti,ab,kw OR (Intraperitoneal Chemotherapy, Hyperthermic):ti,ab,kw OR (HIPEC):ti,ab,kw OR (Hot Chemotherapy):ti,ab,kw OR (Chemotherapy, Hot):ti,ab,kw OR (Intraperitoneal Hyperthermic Chemotherapy):ti,ab,kw OR (Chemotherapy, Intraperitoneal Hyperthermic):ti,ab,kw OR (Hyperthermic Chemotherapy, Intraperitoneal):ti,ab,kw OR (Intraperitoneal Hyperthermic Chemotherapies):ti,ab,kw | 1171 |
| #10 | #8 OR #9 | 1171 |
| #11 | #7 AND #10 | 228 |
